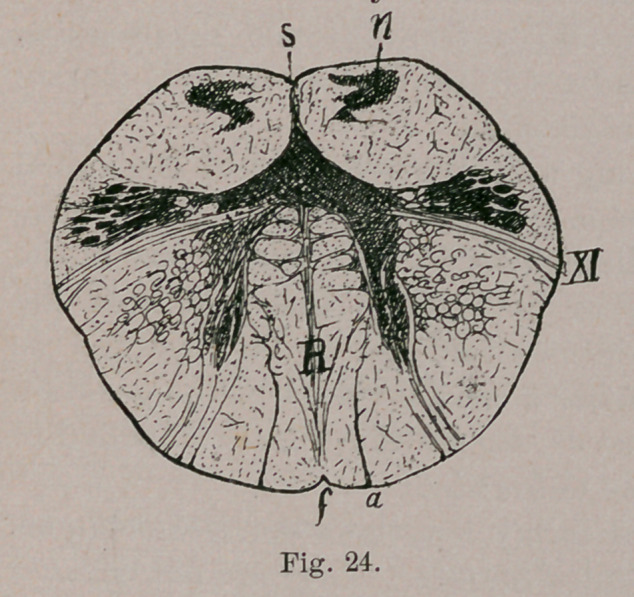# The Comparative Anatomy of the Pyramid Tract

**Published:** 1886-01

**Authors:** E. C. Spitzka

**Affiliations:** New York


### THE JOURNAL

OF

### COMPARATIVE MEDICINE AND SURGERY.

VOL. VII. JANUARY, 1886. No. 1.

### ORIGINAL COMMUNICATIONS.

### Art. I.—THE COMPARATIVE ANATOMY OF THE PYRAMID TRACT.

BY E. C. SPITZKA, M.D., NEW YORK.

#### Introductory Remarks.

The physiological structure of the brain is characterized by the union of two forms of nerve tissue: the *ganglionic* and the *conducting*. The former is the active agent in the reception, registration and association of impressions, as well as the evolution and co-ordination of movements; the latter plays the more passive part of a pathway conveying impressions *to*, or impulses *from* the ganglionic substance. It is the termination of the conducting paths, that constitutes the most accurate test of the functional assignments which are being made to the ganglionic brain centres, of which so large a number have been determined by a combination of anatomical, physiological and pathological methods. The mere knowledge of the names and situations of eminences and depressions of the brain surface is as unprofitable as would be the inspection of the outside of a clock-case to one desirous of understanding the mechanism by which the hands move round the dial. It is the deep structure of the various segments comprising the cerebral mechanism which is of importance to the physician, for in its disturbance the intrinsic features of cerebral symptomatology are to be found.

108216

Physiologically speaking, the brain when separated from its peripheral connections is inert. It is by virtue of efferent and afferent nerve-tracks establishing, as they do, connections with other parts of the body, that the brain becomes the organ of sensorial reception, the seat of automatic and volitional co-ordination, and through these, in turn, the laboratory of the mind. Every intelligent study of the brain must start from this fundamental fact, and it is therefore the task of the anatomist to unravel the paths by which functional impulses travel *to* and *from* the various centres included in the brain. Thus, if the fact be established that a given nerve bundle is connected with the cells related to the anterior roots of the spinal nerves—which, as we know, lead to muscles—it is to be inferred that the gray matter in which such a bundle originates must have a functional relation to motor action. Again, if one end of a nerve-bundle can be traced to the optic nerve, it is reasonably certain that it conveys visual impressions, and that the gray matter with which its other extremity is united must be a visual centre.

The means employed by modern investigators to disentangle the great labyrinth of nerve-tracts in the brain and spinal cord are the following:

I.—The direct examination of nerve-tracts with the naked eye. This method, followed by Gall, Reil, and recently improved by Stilling, the younger,* depends on the tendency of brain tissue, hardened in alcohol, when torn asunder, to break in the direction of the fibre-tracts running through it. Although admirably adapted to illustrate salient features to beginners, little dependence is placed by modern authorities on this method.

II.—The demonstration in microscopic sections of the connections of individual nerve fibres with nerve cells. In prop-

---

* The method of defibrillating brains hardened in alcohol, inaugurated by Gall, and cultivated by Foville, Arnold and Meynert, had, until recently, fallen into disuse. It is certainly well calculated to exhibit the coarse nerve strands in the hemispheres, and even such compact bundles as those of the pyramid tract, where it courses through the pons. Some of the earlier notions about the lemniscus (Reil) were quite correct, though based on this coarse method. Stilling, the younger (Investigations on the Structure of the Optic Central Organs. Part 1: Chiasma and Tractus Opticus. Kassel and Berlin: 1882) claims that the objections urged against the use of this method no longer exist, as the fine sections made by the improved microtomes of to-day, which were not at the disposal of earlier investigators, may erly prepared specimens, the so-called axis cylinder processes of ganglion or nerve cells, may be traced into medullated nerve fibres. In this way the connection of the pyramidal cells of the cortex with the fibres of the corona radiata is readily shown. But what we chiefly owe to this method is the knowledge of the so-called cranial nerve nuclei: small accumulations of nerve-cells at or near the floor of the fourth ventricle, into which Stilling the elder, Meynert, Deiters, Dean, Duval and Clarke traced the roots of the last nine cranial nerves.

III.—The comparison of the relative development of nerve-tracts and centres in animals having peculiar physiological characters. As the development of an organ increases with its function, it is fair to assume that the centres and tracts devoted to a special function will vary with the importance of that function in special animals. Numerous and important facts have been established through a comparative study of the mammalia. The mole, for example, has rudimentary eyes, the consequence is that the anterior pair of the corpora quadrigemina with the connected optic tracts, are also rudimentary. The elephant has a powerful trunk, subservient to a remarkable extent to his intellectual needs; in harmony with this fact, his facial nerve and nucleus which control its motions are unusually large. The bat has overgrown anterior extremities, which are developed into large and powerful wings; correspondingly, the cervical swelling of the cord is very large, while the lumbar swelling is comparatively as insignificant as the hind extremities of the bat are diminutive. In the kangaroo, on the other hand, where the relative preponderance of the extremities is reversed, the relations of the cord swellings is the opposite: the lumbar swelling far exceeds the cervical swelling, just as the hind extremities exceed the anterior. In most cases, where a careful examination has

---

be used as topographical and histological guages. One advantage of the defibrillation method is that it exposes nerve-bundles in their continuity, follows all their undulations, and thus furnishes a plastic conception of their relations in space, which is very difficult to obtain from a study of sections. Identical considerations had been advanced several years before by the author ("Contributions to Encephalic Anatomy. Chicago Journal of Nervous and Mental Diseases.")

Stilling has suggested some refinements of *technique* which, if they accomplish all he claims, are destined to revolutionize the methods now in vogue. For the present his method as well as his published results must be regarded as *sub judice*.

been made, such physiological atrophies and hypertrophies of special peripheral organs have been found to be accompanied by corresponding enlargement or shrinkage of the related nerve roots and nerve nuclei. The comparative method thus constitutes a valuable criterion of the physiological bearing of structural peculiarities.*

IV.—The observation of the course followed by that secondary degeneration which ensues after the destruction of nerve centres, or the interruption of nerve tracts. When disease destroys ganglionic tissue, or interrupts the physiological connection of a ganglion with its naturally associated end organ, the nerve tract connected with the diseased or atrophied ganglion, degenerates.† The precise position of that nerve tract is easily determined by the change in color and consistency which it exhibits. This is the most reliable and valuable method of studying the course of nerve tracts.

In case the animal in whom disease or injury of the brain occurs, be very young, the affected nerve tract is not merely changed in color and consistency, but it may fail to be developed, or having developed, disappear entirely. Thus, by destroying the so-called motor zone of a newly-born cat or dog, the pyramid tract is found to be absent. On extirpating the eye of another, that part of the brain which is connected with the sense of sight remains undeveloped or undergoes shrinkage. We are enabled by this method, which is known as the "atrophy method," to artificially arrest the development of special centres in the brain and cord. Nature, however, often effects the same purpose. Animals are born with an eye or a limb absent; correspondingly the nerves, normally

---

* Exclusive reliance on the comparative method might lead to grave errors. In general zoölogy it would be faulty to argue from the external resemblances of animals that they are related forms, and many popular errors are due to this fallacious manner of viewing things. The whale is a fish—the bat, a bird—the armadillo, a tortoise—the snake, a worm, according to this. Similar errors are due to the superficial study of surface resemblances of the brain. Already one of the ancient writers argued that the brain could not be the seat of the mind, because the ass had as well convoluted a brain as man.

Only related forms should be compared. It happens that, with the possible exception of the elephant, the porpoise is the only animal—besides man and the monkey—in whom the island of Reil is completely covered. Now, the island of Reil is in man, with its environs related to the function of speech. Would it then be just to argue that the porpoise had a high potential of speech faculty? No. The development of gyri in the porpoise's island, and its concealment from view, are evidences of nearly as high, if not equally connected with such parts, are absent, and the nerve tracts and centres in the brain which represent their functional activity, are equally wanting. Waller, Türck, Bouchard, Charcot, v. Mannkopf, Meyer, Homen and v. Monakow have contributed to the knowledge of nerve tracts in the human brain by these various methods, while their course in lower animals has been elucidated chiefly by v. Gudden and his pupils. The first researches of this nature in this country were made by the writer.

V.—The study of the progressive development of nerve tracts in fœtal and young animals. Foville and Meynert discovered that certain nerve tracts in the brain and cord, which are originally equally gray, become white in color (through the acquisition of myeline) at definite periods of fœtal life. It thus becomes possible to distinguish them by this chronological difference in development. But such observations were disconnected and fragmentary prior to Flechsig's researches. It was shown by this author that the study of the myelinic maturation of nerve tracts was destined to clear up the labyrinth of intra-cerebral and spinal nerve tracts far more extensively than all other methods used up to the time of the appearance of his great work.

In the case of several of the better-known brain tracts, the extent to which the various methods confirm each other is striking. They not only sustain each other, but are in accord with the results of empirical physiology and pathology. Thus it is established that the electrical irritation of certain fields of the brain-surface provokes movements of the face and extremities on the opposite side of the body. If now these same fields be destroyed the animal is found to have lost the

---

high brain development *in general.* In man, the island is high (dorso-ventrad) in front, and tapers backwards ; in the porpoise it is the reverse ; it shows its greatest height near the auditory field, which preponderates in the Cetacæ, who have the finest hearing faculty and the largest auditory nerve in the animal kingdom.

In this connection I desire to express my concurrence in a statement made by Wilder before the American Neurological Association. I had stated that the porpoises' island was as large as the human. Wilder finds that in absolute linear dimensions this is true, but that as the human island is very convex and the porpoise's, flat, the surface area in the former may be greater. At the same time, the statement that the porpoise has the most numerous gyri remains unchallenged.

† While it is generally supposed that nerve-tracts degenerate in the direction of the functional impulses which pass through them, there are some remarkable exceptions to the rule that motor-tracts degenerate in a centrifugal and sensory tracts in a centripetal direction.

power over the same parts. Examining the brain, months after the removal of the motor field, it is found that a tract of fibres in the white substance has undergone secondary degeneration, and can be traced by the ensuing color change through the *crus*, *pons* and *oblongata* of the same side, then across, *via* the decussation of the pyramids to the opposite side of the spinal cord. On comparing the brains of young and fœtal animals of the same species it is found that this very tract obtains the white color indicating maturity at a special period. Defective development of the motor region is accompanied by a defective condition or absence of the same tract, and it is possible to produce—create, we were about to say—an animal lacking the pyramid-tract of one side by destroying the corresponding motor province of the cerebral cortex in early life.

If further evidence were wanting to demonstrate the functional and morphological position of the will tract, comparative anatomy would furnish it; just as the cerebral hemispheres preponderate in man, so the pyramid tract is more voluminous in him than in any other animal. On comparing a series of animals, beginning with the marsupials, we find that just as the cerebral hemispheres appear larger as we pass from the lower to higher forms, so the internal capsule becomes more distinct, the contour of the *pes pedunculi* bolder, the pyramids more salient, while the area of the posterior division of the lateral columns, into which the latter decussate, is increased. These anatomical and physiological facts harmonize so completely that we cannot do better than commence our analysis of the cerebral mechanism with that important and clearly understood set of centres and fibre tracts which are concerned in the central regulation of voluntary motion.

As the higher centres of motion are located in the cerebral cortex, and the lower centres are arrayed in a nuclear series extending from the aqueduct down to the terminal cone of the cord, while the connecting tracts occupy important positions in almost every segment of the nervous axis, we shall throw a rapid glance at the morphology of the brain and cord as a preliminary.

The central nervous system precedes all other organs of the body in development. It is the first permanent structure indicated in the mammalian germ, in whose shield-shaped area (Fig. 1)

FIG. 1.

Area Embryonica of rabbit. The upper streak is the medullary lamina, the disappearing primitive streak, which is a temporary structure, and vanishes as the medullary streak is developed, is indicated at the narrower or caudal end of the shield. As the specimen was drawn by reflected light, the thicker part of the germ, both on its margin and in the axial structures, is indicated by clear spaces.

it occupies a dorso-axial position, to be retained ever after in the maturing body. The cells of the germ which are the products of the yolk-cleavage following fertilization, are closely crowded in the median part of the upper of the three layers (Fig. 2) of which the germ consists. They present to the eye an opaque appearance, like a streak extending from the head-end to the tail-end of the otherwise transparent *area* of the embryo. This streak, known as the medullary lamina, is the first trace of the brain and cord.

The cells of the medullary lamina continue to preponderate in growth, and soon those at the edges rise, including between them a depression, concave from side to side, and known as the medullary furrow. As its edges continue rising they approach, and finally coalesce, completing the closure of the furrow, into a medullary canal. The process by which the flat lamina become furrowed, and then converted into a tube may be compared to the rolling up of a strip of tin to form a pipe.

Fig. 2.

Diagrammatic trans-sections to illustrate the three super-imposed cell layers of an early germ, and their foldings in three successive stages: (*e*) medullary groove, closed in the third stage represented, to form the central canal of the cord.

The medullary tube, whose surrounding wall is to form the solid tissues of the brain and cord, while the lumen within is to constitute their natural cavities, is at this stage of nearly uniform calibre. An enlargement is noted at the head end (Figs. 3, 4, 5, 6) which indicates the location of the future brain.

Fig. 3. Fig. 4. Fig. 5.

Diagrammatic representations of three successive stages in cerebro-spinal segmentation.

The brain portion outstrips the cord portion in growth, and early exhibits two constrictions imperfectly separating it into three globular sub-divisions: the primitive vesicles of the brain (Fig. 4). Each of these vesicles contains a dilated subdivision of the medullary canal communicating with the others through narrow orifices, which correspond to the abovementioned constrictions; these cavities are the primitive cerebral ventricles (Fig. 7). In the order in which they lie, the vesicles are spoken of as the fore-brain, mid-brain and hindbrain. The latter is continuous with the cord, which remains the longest, but also the narrowest segment of the central nervous system. The cavity of the cord becomes narrowed to almost capillary dimensions, and is known as its central canal. The cavity of the hind-brain becomes the fourth ventricle, that of the mid-brain becomes a narrow passage connecting the fourth ventricle with the cavity of the fore-brain, and is hence known as the aqueduct. At first the mid-brain is the most prominent of the brain vesicles, but remaining comparatively stationary, thereafter is outstripped in development by the fore and hind brain, which undergo modellings, resulting in the formation of the most massive of the brain divisions.

Fig. 6. Fig. 7. Fig. 8.

Diagrammatic representations of the same stages shown in the preceding figures, the dorsal walls being sliced away to expose the ventricular cavities.

The fore-brain exhibits a slight indication of a division into an anterior and posterior segment. The anterior is composed of two symmetrical parts, each of which seems as if it were a side-bud attached to the posterior, unpaired division of the fore-brain. These buds are the cerebral hemispheres (Fig. 6), They are hollow; the cavity of each is known as a lateral ventricle, and communicates, through the *Porta monroi*, with the cavity of the unsegmented or "parent" part of the fore-brain (Fig. 8). After the separation of the cerebral hemispheres, with their contained lateral ventricles, the cavity of the parent part is named the third ventricle.

The roof of the fourth ventricle undergoes a thinning out in nearly its entire extent. The most anterior portion constitutes an exception, and becomes greatly enlarged, forming a sort of ledge behind the mid-brain, which is the rudiment of the cerebellum (Fig. 6).

After the formation of the cerebral hemispheres, and the cerebellar rudiment, the brain of the mammalian embryo resembles that of the adult reptile. The hemispheres are so small that they fail to cover the other segments. The mid-brain in both consists of two rounded eminences: the optic lobes. The cerebellum is a simple fringe or cap, covering the anterior part of the fourth ventricle. The resemblance is still more striking in minute details. The chief difference lies in the relative development of the olfactory lobes. In both cases these are hollow extensions proceeding from the cerebral hemispheres, but they are much larger in the reptile, and attached to the front of the cerebrum, while in mammals the insertion is nearer to, or at the base. There is also a great difference in the direction of the encephalic axis. In reptiles, it is comparatively straight (Fig. 9), there is but little deviation of a line drawn along the base of the brain, from the horizontal. In mammals, this deviation is considerable (Fig. 10), greater in the highre than in the lower forms and greatest of all in man. One of these deviations is a little above (cephalad of) the foramen magnum, at about the junction of the spinal cord, and the medulla oblongata. The concavity of this curve is directed towards the base of the brain. The brains of most adult mammals exhibit a marked impression, running transversely on the basilar-face of the oblongata, which I propose to designate the *basilar bend.* It has never, to my knowledge, been referred to, and never been named, but it is clearly in many figures of the embryonic human brain, and in Figure 2 of Plate 1, of Wilder's monograph on the "Brain of the Cat."

Fig. 9.
Lateral view of brain of Logger-head Turtle. (*Thalassochelys Caouana*).

The next deviation is in the region where the Pons is to develop. Its convexity rests on the floor of the skull. It does not interest us here. The third, and main, deviation is, as it were, a reaction from the Pons curve, and is in the opposite direction. It is due to its greater extent in mammals, and its lesser extent in birds, that in the latter, the cerebral hemispheres, when developing, do not over-lap the mid-brain (optic lobes), but push their way between them to come in direct contact with the cerebellum. In this way the optic lobes become crowded to one side, and eventually lie at the base of the bird's brain, a position which they occupy in no other amniote vetebrate.

Fig. 10.
Lateral view of brain of embryonal dog.

In the mammalia the cerebral hemispheres gradually overlap the other segments of the brain. It is exactly as if the parent part of the fore-brain, the mid-brain and hind-brain constituted a stalk, to one end of which (the fore-brain) two buds (the cerebral rudiments) were attached. The buds, however, grow and grow, till they press against each other, and then, gradually swelling out backwards, eventually hide the stalk from view (Fig. 11). The concealment of the various

Fig. 11.

Brain of fœtus, the callosum divided, and hemispheres separated to show the stem (axis).

segments is not, however complete in all forms. In the marsupials (opossum) hedgehog, and small bats—the mid-brain is uncovered; in some rodents (hystrix) the cerebellum is entirely uncovered; in the carnivora, the cerebellum is partially uncovered, being most concealed in the following order, which closely corresponds to the grade of cerebral development: fox, cat, dog, coatimundi, bear, sea-lion. A similar series can be constructed of the agglomerated proboscidea, artiodactyla, perissodactyla and cetacea: hippopotamus, horse, sheep, deer, ox, pig, elephant, porpoise. In the monkey tribe alone (including man) is the entire brain axis, with its cerebellar outgrowth covered by the overgrown cerebral hemispheres, so that looking from above only the latter are seen. The lowest forms, however: the prosimiae, such as the lemurs, have the cerebellum partly uncovered. The following genera have been examined by the writer, and found to correspond to man in this regard: hapale, nyctipithecus, cebus, ateles, cercopithecus semnopithecus (*maurus* and *entellus*), cynocephalus (*babouin*, *olivaceus* and *porcarius*), macacus (*radiatus* and *cynomolgus*), troglodytes (*niger*), and simia (*orang*).

The overgrowth of the cerebral hemispheres may be regarded as an *index* of the domination of the higher nervous co-ordinations known as mind, intellect, etc,, over the reflex and automatic centres situated in the cerebro-spinal axis, and extending from the thalamus to the conus terminalis. The controlling influence is exerted through nerve bundles, which connect the hemispheres with the lower ganglia. The best parallel that can be used to illustrate this important relation of the *controlling* higher centres to the *controlled* inferior centres is that of a well-generaled army operating in the field against a foe: a parallel which is strengthened by the fact that the nervous mechanism is all-important in that contest for existence in which every healthy organism participates. The outer world is stored with inimical influences, on the one hand, and with the objects which the animal requires for its support, on the other. The inimical influences must be avoided if they operate on a weak point, skillfully and energetically repelled, if they cannot be avoided, and the useful appropriated on the line of march through life. The desirable, as well as the inimical influences are recognized by the peripheral end organs of sensation, the attacks of the foe are met by the motor apparatus. The latter is also exerted in obtaining the needful.

In this parallel, the peripheral organs of sensation obviously play the part of *vedettes* who communicate with the reflex battalion chiefs or the nerve nuclei of the spinal cord and brain axis. If the former discover movements of the foe, the battalion chief, through his adjutant (the motor nerve) may hurl his forces (the motor-end organs) at the foe, or withdraw them. In a close attack, indeed, he has no other alternative than those "reflex acts." But as there are a large number of battalions and squadrons in the army who require united management, a field-marshal is present (the imperial cerebrum) who is informed by fleet couriers and field telegraph (the centripetal fibre system) of the reports which each battalion chief receives; who is, from his central position, able to compare and combine the impressions received along the whole line (that is, those coming from *all* the sensory organs in the aggregate) and then intelligently directs advance and retrograde movement by signals (through the centifugal fibre-tracts). Thus the battalions may be deployed most advantageously, retreating before threatening attacks, whose extent their own vision is of too limited a range to discover, avoiding ambuscades and striking the foe at the weaker points unmasked by their comrades. The eye may see a distant danger which the skin does not feel, and the ear places the brain on guard against a foe coming from some other direction than that covered by the eyes.

It would be manifestly incorrect in this parallel to attribute the exclusive credit for a successful movement, or the blame of a failure, to the field-marshal. If the *vedettes* do their duty; if the battalion chiefs properly transmit their information; if the signals are not interfered with, and the battalions are well disciplined and brave—that is, if the sensory end organs are healthy, the reflex arches, and the conducting tracts uninterrupted, and the muscles under control—intelligent orders given by the commander-in-chief will be properly executed. But if the *vedettes* are asleep, the army may be surprised, or if the messages to or from headquarters are interrupted, the threat, ened detachment may be destroyed without the field marshal's knowledge or responsibility. Thus, the hand may be severely burned without the conscious knowledge of the brain, if the nerves of the brachial flexus be divided, if the cervical enlarge ment of the cord be destroyed, or if the sensory impressions coursing in the internal capsule be intercepted by hemorrhage, softening, or tumors. Just as the battalion chief is a necessary, though humble, agent in the movement of an army, so the reflex centres are essential to the acquirement and execution of functions on the part of the higher centres.

From the foregoing, it is evident that the centres for the individual functions of the periphery are multiple, and differ as to grade; there are centres of lower, higher and, in some cases, of intermediate physiological dignity. In the case of the muscles which move the eye-balls, there is one set of centres for each of the individual muscles in the "nuclei," situated near the Aqueduct of Sylvius, and the neighboring ventricle cavities; there is a higher centre in the optic lobes, which coordinates these individual muscle centres, in *unconscious* union, subservient to retinal impressions; and this automatic mechanism is in its turn under the dominion of a cortical area in the organ of intelligence, which employs it in the *conscious* direction of the eyes to objects in which an intelligent interest is taken. In this and analogous mechanisms, the various centres connected with a common periphal organ are not strictly independent of each other. The higher centres utilizes the channels and energies of the lower. Thus, the reflex, the automatic and the intelligent impulses to movements of the eye all pass through one and the same oculomotor, abducens, and trochlearis nerves, and one and the same nuclear connections of the nerves.

With these preliminary observations we are prepared to examine the comparative development of the pyramid tract in different species of animals.

### I.—Surface Relations.

The term *Pyramids** is given to two symmetrical eminences of the ventral (*inferior*) face of the brain. They extend from

---

* They were sometimes designated as *anterior* pyramids, in contradistinction to the slender columns of Goll, which were termed posterior pyramids. The latter term being now abandoned, the prefix is unnecessary.

the caudal (posterior) border of the *Pons* to the bend* of the *oblongata.* Up to the present day, the greatest confusion prevails among comparative anatomists regarding the relations of these bodies in the lower mammalia. The fact, that in man the columnar eminences of the pyramids enclose between them the ventral fissure of the oblongata, has determined the designation of every eminence in any mammal, bordering on this fissure, as a pyramid. This superficial procedure has led to the attributing large and well developed pyramid tracts to the very animals which have not the slightest, or at best, but rudimentary traces of them. To-day we are not content with deducing analogies from mere surface resemblances, and inasmuch as the pyramids of the human brain represent the surface development of a part of the centrifugal course of that nerve tract which mediates the voluntary movements of the extremities, it behooves us to be careful how we attribute well-developed pyramid tracts to animals who have corresponding eminences, but who have atrophic or rudimentary extremities.† But even a consistent and critical surface study of the oblongata would have sufficed to establish one of the most interesting criteria of the zoölogical position of certain mammalian groups. To deserve the designation pyramids on the ground of resemblance to the corresponding columns of the human brain, the following conditions must be complied with (Fig. 1):

1st. They must be columnar.

2d. They must appear to emerge from the substance of the Pons.

3d. They must lie entirely ventrad of the *trapezium.*

---

* This is a characteristic feature of the oblongata, and a remnant of one of the embryonal curves. Although not designated by any special name, it has undoubtedly been noted by many observers. Wilder figures it very early and accurately in his lithographic plates of the cat's brain (Anatomical Techology.) It is most distinct in those mammals in whom there is an abrupt decrease in the dimensions of the oblongata towards the cord, such as the bat, rodentia, artiodactyla, in lesser degree, the zona-placentalia, and least of all, in adult man. In the human embryo it is very well marked, and probably the acuteness of the inflection is not an unimportant factor in the causation of the decussation of the pyramids.

† See the reference to Serre's argument in the sequel.

4th. They must, if there be visible olivary eminences, lie mesad (within) of the latter.

Fig. 12.

As far as such columns can be distinguished in mammalian brains, those of all the zonaplacentalia, the anthropomopha artiodactyla, and several other groups to be spoken of comply with these conditions. In the proboscidea as well as the cetacea, there are no bodies which comply with even one of them. No columns are seen bordering the ventral or basilar fissure, which emerge from the Pons. A transverse section through the caudal half of the latter shows that it is made up of gray substance and transverse bundles exclusively. No sagittal fibres exist. It is true that both in the elephant and the porpoise two symmetrical eminences enclosing the median sulcus* are found situated a short distance behind the caudal border of the Pons. But these eminences have the following distinctive characters:

Fig. 13.

Trans-section of Pons Varolii of elephant, showing absence of sagittal (pyramid) fibres in Pons.

1st. They are not columnar, but oval, being distinctly rounded in front and behind.

* This is not a deep fissure, as in some animals.

2d. They do not appear to emerge from the substance of the Pons; there is a deep trench * between them and the latter; microscopic sections reveal the absence of any corresponding continuation in the substance of the pons proper.

3d. The trapezium not being exposed in the elephant or porpoise, does not come in contact with these bodies, inasmuch as they have no relation with the concealing ledge of the Pons.

4th. Microscopic section shows these bodies to be the *true* olivary nuclei.† It is the absence of the pyramids that accounts for the coming in contact of the olivary eminences, just as in human monstrosities where the pyramid tracts are absent, the olives become entirely exposed, and lie together on both sides of the basilar fissure. (Rohon.)

From the above, then, the following proposition‡ may be advanced:

The Proboscidea and Cetacea are characterized by the absence of pyramids in the oblongata, and the consequent exposure and mutual approach of the olivary eminences.

---

* This is most marked in the elephant.

† Meynert remarks ("Klinik der Krankheiten des Vorderhirnes,' p. 27): "The olives of the aquatic mammalia were already known to Stannius." It is everywhere evident throughout Meynert's writings that he has utterly failed to appreciate the fundamental distinction between the *phocidæ* and the *cetacea*, and confounded them under the common designation "aquatic mammals." Burdach, in 1819, discriminated, he says: "Only with dolphins, seals and apes are they (the olives) more developed. Still much less so than in man." He seems to rely on Stannius, Treviranus, Carus and Tiedemann for the substance of this statement.

‡ The discovery that these animals have no pyramid tract was made by Spitzka, and announced, as far as the elephant is concerned, in the following papers: "The Sensory Tract of the Fore-Brain"—*Chicago Medical Review*, July 5th, 1881. "A Contribution to the Morbid Anatomy of Pons Lesions" *American Journal of Neurology*, Nov., 1883. The condition of the parts in the porpoise, while referred to in earlier communications, was first distinctly asserted in "Contributions to the Anatomy of the Lemniscus,"—*N. Y. Medical Record*, Oct., 1884. It is singular that this should not have been noticed before, and the more so as it must have been difficult for Stannius and other older observers to recognize the large size of the ordinary eminences, without, at the same time, noting the absence of the pyramids in the porpoise. Even the accurate and painstaking Lockhart Clarke speaks of the "pyramids" of both animals, but he does this rather on the strength of the statements of others.

This adds another ground to the numerous reasons* advanced for classifying the Cetacea near the old group of the Pachydermata, represented in modern classification by the proboscidea, perissodactyla, and non-ruminant artiodactyla with the possible inclusion of the sirenia.

But aside from the signification of this proposition to the zoologist, it furnishes much food for reflection to the physiologist; it does away with the dictum of Meynert that the development of the pyramids is one of the indices of cerebral, *i.e.*, intellectual development. † Large pyramids do not signify a large cerebrum, and small pyramids a small cerebrum. The elephant and porpoise have both far more richly convoluted brains than man. Whatever guage of cerebral *status* be applied, they will be found to occupy a very high position. They approach the anthropomorphæ in the almost complete concealment of the cerebellum (porpoise), in the complete concealment of a large, well-convoluted Island of Reil, in the surface dimensions of the Pons, and in the presence of a posterior ventricular horn, as well as in numerous other coarse and histological features. Their intelligence is of a high order.‡

---

* Beauregard ("Recherches sur le Cerveau de Balænoptera Sibaldii; Société de Biologie Communiquée le 10ieme Fevr., 1883), finds remarkable analogies between the gyral type of the whale and the horse, and calls attention in this connection to the fact that the urogenital apparatus, as well as the placental relations of both groups exhibit a close relationship.

† This dictum was originally, I think, advanced by Gall, and with the limitations to be suggested, has some force. Even so old a writer as Burdach hesitated to accept it unreservedly, while Lockhart Clarke appears more prepossessed in its favor. Meynert elaborated the suggestions and facts of his predecessors, and the theories are customarily ascribed to him. It is adhered to in his latest publication (*op. cit.*)

‡ I proposed some years ago that the area of a trans-section of the spinal cord at the level of the foramen magnum be taken as a unit standard of comparison, and the average between the trans section and flat-section of the cerebral hemispheres expressed in such units. This would give an approximate idea of relative cerebral development in different animals. Or the cubic area of the brain might be expressed in cubes of such a unit. Either method would be physiologically more just than the comparison of brain weight and body weight.

Contrary to what many believe, the porpoise is an animal of exquisite sensibility, skilled motor endowments aud a high emotional nature, as well as possessed of considerable judgment. The reader will find some facts pertaining to this in another part of the article.

The absence of pyramids in these animals shows that intellectual (voluntary) motor control may travel through other nerve tracts than the pyramids. I shall recur to this special question after completing the survey of the surface character of the so-called pyramids in other of the animals examined by me.

### II.—Historical Observations.

As the history of the discovery of the anterior pyramids and of the various views announced about their relations is not the last interesting chapter of this subject, and some of the errors of the older, and even some modern, writers, are quite instructive, I will briefly mention them here.

The anterior pyramids were first noticed by Eustachius, but Willis described and named them. Malacarne found that they become narrower towards the cord, and gradually disappear from the surface. At this period only the surface features of the brain were well studied, and the substance of the knowledge of the day may be summed up as follows: In man, the pyramids (*corpora pyramidalia antica*) are two columnar elevations, bordering the basilar fissure of the medulla oblongata, large and prominent at their cephalic end, they become lost under the posterior border of the Pons, while their narrow and sharply-pointed caudal ends dip into the fissure which lies between them, and which they border.

It was Mistichelli who discovered that the fibres of which the pyramids are composed decussate (that is, cross into the opposite side of the spinal cord), and he recognized, nearly two hundred years ago, the relation of this fact to the well-known observation, that in paralysis, from cerebral apoplexy, the paralysis is on one side of the body.* His description was inaccurate in detail. It was corrected in part by Santorini, but succeeding writers contested or doubted the existence of a decussation till Gall forever set the question at rest and demonstrated its coarse relations very clearly. By him it was supposed that each pyramid crossed the median line as a totality, but Rosenthal first asserted that while the greater part crosses into the opposite half of the cord, a small portion remains on the same side. This view is the one accepted at the present day. To the same author belongs the credit of

* Opposite to that of the disease.

having shown that the decussating part of the pyramids is derived from the opposite lateral, and not the anterior, column of the spinal cord, as had been supposed by his predecessors.

Lockhart Clarke, who also discussed the historical aspect of the question, appears with Serres, whom he cites, to have been the first to study the comparative anatomy of the pyramids. He recognizes that, while as wide or nearly as wide in lower mammals as in man, that they are of equal depth in no animal. He says: "They are also of considerable, but variable, size in all the mammalia, and in some of the larger beasts of prey, as the lion, hyena and jaguar, I have found their external dimensions to be nearly, if not quite, equal to those of man, but in depth they are smaller among this class of animals. Well-marked, prominent and rounded in the quadramana—at least in the simiadæ—they are broad and flat, but usually larger in the herbivora, and in the elephant, though far from proportionately broad in relation to the size of the brain, they are long and tapering. 'According to the hypothesis,' says Serres, 'that the pyramids are the roots of the cerebral lobes, their development ought to be proportionate to them; now, this is not the case; the pyramids are less bulky in man than in the apes, and they are much more pronounced in the cetacea. This predominance of the pyramids extends also to the carnivora—the lion, bear, weasel and others; to the pachyderms and ruminants.' In relation to the size of the cerebrum, it is true that the anterior pyramids are smaller in man than in some of these animals, particularly the larger carnivora, such as the lion and hyena; but actually their dimensions are in some instances much greater. In the herbivora especially the superficial breadth of the pyramids is no indication of their actual bulk, which can be ascertained only by means of transverse-sections; for although they are frequently very broad, and *rendered prominent at one part by the olivary bodies behind them* (italics ours), they are much more shallow than in man, *and their decussating fibres are much less numerous* (italics ours) * * * * * " Elsewhere he says of the decussating fibres of the pyramids, "I have found their number to be in the direct ratio of the *prominence*, rather than the breadth of the pyramids. They proceed more from the *deep* strata of the lateral column, and less from the *superficial* and

*anterior*, so that the whole decussation, as will be seen later on, resembles rather the *upper* portion of that in man." "In the cat it is stronger than in the dog, sheep or ox, and a transverse section through it presents a very beautiful appearance. A transverse section of the medulla oblongata of the tiger bears, in every respect, a most striking resemblance to that of the cat, the decussating fibres are numerous, and the anterior pyramids are consequently prominent and deep, so that relatively to the size of the cerebrum they are decidedly larger than in man. In the guinea pig, also, the decussation is well-marked, and the fibres it derives from the posterior columns and the gray substance may be very distinctly seen."

It is evident from these citations that Clarke was on the threshold of making an important discovery, one that would have been of value to the classifying zoölogist as to the human and veterinary pathologist. He was unfortunately hampered by the now demolished theory that the fibres which decussate from the nuclei of the posterior column in the fir-cone deccussation, to form the inter-olivary layer, are a part of the pyramids. His ingenious observation that the "decussation (in herbivora) resembles rather the *upper* portions of that in man," is to-day to be read thus: Only the fir-cone decussation (piniform deccusation) is present to any noteworthy extent in the hoofed animals. Clarke also saw the door open to the conclusion which we shall advance as to the relation between prehensile function and the pyramid tract, when he noticed that the cat and tiger had nearly as large pyramids as man. But he went no further than the observation, and drew no conclusions from it.

With regard to the singular argument of Serres, cited, the making of transverse sections (advocated with so much reason by Clarke) seems to have been neglected by him. Since he, in consequence of this, mistook the olives for pyramids, his objection to the theory, sustained by most writers, falls to the ground. In addition, he was misled by the luxuriant arciform cingulation of the human oblongata, inferring that the pyramids were, *de facto*, less prominent than in the apes. In truth, it is the reverse.

Dean*, the only earlier American writer who has devoted

* The gray substance of the medulla oblongata and trapezium.

much attention to the subject, says, in speaking of the changes undergone by the cord as it passes into the oblongata, "The pyramidal columns which, in the sheep are very small, are now quite distinct, and numerous fibres run parallel to the axis of the medulla, forming with the unciform fibres which decussate with them the raphé," In speaking of the olivary relations, he says: "In the cat, and carnivora generally, the anterior pyramids are deeper, and approach more nearly to the human type than the sheep's."

In this connection, I think it but justice to Solly to state that although writing in 1847, he was possessed of a knowledge of all the facts relating to the olives above cited from Clarke and Dean. Indeed, he seems better than any other early writer, to have recognized the real source of the discrepancies among authorities (*vide* pp. 107, 108): Longet says ("Anat. et Phy. du Systeme Nerveux de l'Homme et des Animaux Vérté-brés," p. 390), "The olivary bodies attain their highest devel-opment in the human species. It is often impossible to per-ceive these eminences in other mammalia." "Having carefully examined," says Rolando, "the place where these eminences ought to be, I can assert that they are not to be met with in the ox, pig, sheep or goat." Carus affirms that they are wholly absent in most mammalia, or at least, that they do not present the arborescent appearance of white and gray neurine which they do in man. "I have found these ganglia in the sheep, horse, calf and cat, and I have no doubt that they exist in all the mammalia." "Gall," says Longet, "has certainly exagger-ated their volume in the calf; they are sufficiently apparent in the ape, but especially apparent in the porpoises and in the dolphins." In the sheep the *corpora olivaria* do not project on the surface. They are best detected by a transverse section; and they will be found not on the side of the pyramidal bodies, but behind." Solly is, however, very vague about the relations of the pyramids and trapezium. Thus in Fig. 51 (base of sheep's brain) he represents the trapezium crossing bodily so as to entirely conceal the pyramids. In Fig. 55 (base of the horse's brain) he represents the lines of the late-ral pyramid boundary, and of the posterior border of the tra-pezium crossing each other. One of these lines, of course, must be fanciful. The sketch of the base of the elephant's brain, evidently made by the author himself (Fig. 57) represents magnificent olivary protuberances and pyramids, side by side. Either elephants differ among themselves beyond the customary range of variation, or the draughtsman labored under illusions. Probably Owen, whose description of this brain Solly cites, interpreted it more correctly, for he speaks of the large size of the *corpora olivaria*, and says nothing of the pyramids. Solly correctly appreciates the enormous development of the olives in the porpoise, but he makes this singular interpretation. "The corpora olivaria are so amazingly developed that instead of being separated from each other, as in the human being, by the corpora pyramidalia, they *overlap and cover* these so completely as even to come into contact with each other, in the median line." They can come into such contact only in the absence of the pyramids.* Solly probably fell into this error in attempting to reconcile Leuret's and Tiedeman's figures.

In another place (pp. 183, 184) Solly discloses a fact which, if sufficient stress had been laid on it, and if his work had been less ignored, would have rendered this discussion unnecessary. "In the sheep, the horse, the cat and calf, I find that there is a wavy line of gray matter in the very centre of the corpus pyramidale, which is clearly the analogue of the corpore olivaria in man." He fails, however, to see that this fact opposes the existence of a true pyramid in these animals; on the contrary, he utilizes it to prove that the olives and olivary tracts have motor relations. Besides, his statement is incorrect as far as the cat is concerned.

How little advance has been made since the days of Solly? Bastian, who by-the-by, "lives on" Solly, as far as the anatomical portion of his work† is concerned, does not seem to

---

* As in human monsters whose pyramids are absent, in consequence of non-development of the hemispheres.

† "The Brain as an Organ of the Mind," 1880. This book has had the unmerited honor (like Beard's "Creation") of a German translation. In neither case, I think, reflecting favorably on the discriminating power of German publishers. In this connection, I desire to correct an erroneous reference in my article on the Corpora Quadrigemima ("Mitheilung die angebliche Abwesenheit der Vierhügeltheilung bei Reptilien Betreffend Mendel's Centralblatt," 1884) I there attributed a figure of the boa's brain to Solly, it really being Fig. 58 of Bastian, and is taken by him from Rhymer Jones after Swan. It is only approximately correct in regard to the corpora quadrigemima.

have been able to master even the correctly given details furnished by his predecessor. He figures a dolphin's brain, from Owen, after Tiedeman (Fig. 74) in which the very bodies, correctly designated as olivary by Solly, are termed pyramids, being sufficiently distorted to merit that title.*

Aside from a few disconnected observations made by Stieda, I can find nothing else on this head,† excepting the important cautionary statement of Flechsig relating to the brains of the muridæ, and to which I shall have occasion to direct special attention.

### III.—Comparative Characters.

I have myself examined a large number of brains, and noted the peculiarities of the pyramids in them. In order that none of the conclusions advanced in the following pages may be wrongfully supposed to apply to forms allied to those examined by the writer, but not now at his disposal, I append a complete list of them, arranged in their zoological order.

### PRIMATES.

Anthropidæ : Man.

| | | |
|---|---|---|
| Simiadæ | Anthropomorpha | Orang. Chimpanzee (three specimens). |
| | Cynomorpha, Old World Catarrhine Apes. | Semnopithecus entellus. |
| | | " maurus. |
| | | " mitratus. |

* In Solly's figure (from Leuret) there is some indication of a remarkable body connected with the auditory nerves, and which in structure, at least, does not appear to be the trapezium, but a mingled ganglionic and fascicular mass related to those nerves, which, as known, are larger in the porpoise than in any other examined mammal. Gross and inexcusable errors disfigure Bastian's text. Thus, he speaks of the corpus trapezoideum as concealing the olivary bodies from view in lower animals, and shrinking cephalad to expose them in higher—thus showing that the primitive discrimination between the nucleus of the trapezium (upper olive) and the true olive, was not at his disposal. The condition of medical book reviewing to-day may be inferred from the fact that but a single review of the many calls attention to the numerous similar errors in this work.

† Among those who have devoted attention to comparative cerebral anatomy, it is the veterinary anatomists above all who have evinced the least originality and criticism in observation. Indeed, they seem to have accepted the description of the human brain as applicable to that of the horse, attributing to it the same nerve tracts, ventricular features, etc. Occasionally one of these authors, rather than mislead his pupils, in the absence of any positive convictions, leaves his descriptions and, occasionally, the accompanying illustrations so obscure and ambiguous, that it is left to the student to draw his own inferences. To illustrate this, I need but point to the plate of the base of the horse's brain, and the manner in which the boundary lines of trapezium and pyramids intersect in McFayden's book, and this is a superior one of its class.

Cercopithecus griseo-viridis.
Cynocephalus babouin.
" porcarius.
" olivaceus.
Macacus radiatus.
" cynomulgus.
" nemestrinus.
" rhesus.

{Platyrrhini, New World Apes.} {Ateles melanochir. Nyctipithecus trivirgatus. Cebus capucinus.}

{Arctopithecini, Marmosets.} Hapale jacchus.

LEMURIDÆ : Arctocebus calabarensis.*

### CHEIROPTERA.

(Frugivora) Pteropus fuliginosus (5 specimens).

{Insectivora} {The common Austrian species. " " New York species.}

### INSECTIVORA.

Scalops Americanus.*

### RODENTIA.

Lepus Americanus.
" cuniculus.
Cavia cobaya.
Cælogenys paca.
Sciurus.*
Hystrix Canadensis.
Mus ratus.
" musculus.
Castor fiber.*
Arctomys monax.

### CARNIVORA.

FISSIPEDIA : Canis familiaris.
" vulpes (Americanus)

---

* I take pleasure in recording my obligations to Dr. William A. Conklin, of the Zoölogical Department of the Central Park Menagerie, and Mr. Henry Reiche, proprietor, with Dr. Dorner, the Superintendent of the N. Y. Aquarium, for the majority of the rare animals enumerated in this list. In addition, I am indebted to Dr. E. G. Messemer for the brain of one black bear, to Dr. E. C. Seguin, for that of a rhinoceros, to Dr. Miles, of Baltimore, and Prof. Osborn, of Princeton, for several opossum-brains; to Dr. H. G. P. Spencer, of Watertown, two porcupines; to Dr. E G. Rave, of Hicksville, for that of a woodchuck, and to the exertions of Mr. Cohn, formerly connected with the Barnum show.

Felis leo.
" leopardus.
" catus.
Ursus Americanus.
" (Helarctos) Malayanus.
Procyon lotor.*
Nasua rufa.

PINNEPEDIA: Lutra Virginica.*
Zalophus gillespiei.
Phoca vitulina.

PROBOSCIDEA.

Elephas (Loxodon) Africanus.

UNGULATA ARTIODACTYLA (RUMINATIA).

Cephalophus mergens.
Cariacus Virginianus.
Capra hircus.
Ovis aries.
Bos taurus.

UNGULATA ARTIODACTYLA (NON-RUMINANTIA).

Sus scrofa.
Hippopotamus.

UNGULATA PERISSODACTYLA.

Equus caballus.

Rhinoceros * (two-horned dark species.

EDENTATA.

Cholopus didactylus.
Dasypus sexcinctus.*

MARSUPIALIA.

Didelphis Virginica.*

On glancing over this collection, I was struck with the presence of two prominent types of pyramid development. These I shall call:

I. The pyramid of pointed and defined termination.

II. The pyramid of diffuse and undefined termination.

---

I might have enlarged the series, if in addition to my own dissections, I had utilized the drawings and descriptions of previous observers. Unfortunately these are, with rare exceptions, unreliable. Clarke, Dean, Huschke, and the much-ignored Gall, alone have correctly represented the base of the brain in several species of animals. Even where the author cannot be suspected of carelessness or neglect, his artist has taken liberties with nature that mar his representations. Thus, one of Gudden's best papers is accompanied by two plates containing no less than five types of pyramid development in one and the same species of animal—the rabbit—one, at least, of which I do not believe is a possible one. Those species represented by imperfectly preserved specimens are marked by an asterisk.

The noteworthy feature of these two types is that they closely follow certain zoölogical limits. The first is found in *all* the Primates and Carnivora. The second is found in *all* the Ungulata. I shall give reasons when discussing the deep structural relations, for regarding the condition found in the cetacea and proboscidea, as an extreme phase of the second type.

The following diagrams illustrate the salient features of both :

Fig. 14.

First Type. Second Type.

The second, or aberrant type, has the smallest pyramids. The brains showing it comply with the requirements of its definition previously referred to, only so far as the cephalic end of the pyramid is concerned, Here they are distinctly columnar, and lie, to the naked eye, at least, ventrad of the trapezium. But towards the cord they are more indistinct.* In

* The apparent exception, offered by the sheep's brain, and to be discussed further on, does not invalidate this rule, for the distinct line in that case is not the ordinary boundary, nor homologous with it.

the first place, the hoofed animals, instead of a ventricular (basilar) fissure, on the greater and caudal portion of the oblongata, have a faint sulcus. Consequently, the demarcation between the two pyramids, which is very distinct in the first type, is but faintly indicated. In like manner, the ectal (outer) boundary, which is marked in the first type something as a steep but low hill is marked against the underlying valley, is, as it were, blurred, and as we shall see, and as Solly discovered, is in the main due to the prominence of concealed structures. In the hippopotamus, indeed, it is impossible to identify the dividing line between the so-called pyramids and the lateral field of the oblongata,* except by means of the nerve

Fig. 15.

Type of the hippopotamus.

roots. In none of the ungulates, that is, the animals exhibiting the second type of pyramid, is a decussation of pyramid

* I refer, of course, to the posterior (caudal) part of the oblongata. In the anterior or trapezial portion there is a slender pyramid pair, of even more attenuated dimensions than in the calf. I may add here that the lateral field of the oblongata in the hippopotamus bears a round, faint elevation on each side, laterad of the pyramid and caudad of the trapezium. Possibly they correspond to the lower facial nucleus. (A, A in diagram).

fibres made visible on separating the ventral fissure of the oblongata, although represented as so occurring in numerous treatises. In the first type—that found, from man down to the fox, including all the primates and carnivores, the "pointing" of the pyramid is accounted for by the dipping of its component bundles into the ventral fissure, to cross the median line at its floor, and enter the deep tissue of the lateral column on the opposite side. It is this crossing (decussation) of the major part of the pyramid tract, which accounts for the fact that when the motor centers or tracts of the brain are injured on one side, the ensuing paralysis is on the opposite side of the body. This is an almost invariable law for all the animals exhibiting the first type of pyramids, for, as stated, they all have a decussation of that tract.

Fig. 16.

I will add, provisionally, that as regards the external relations, the pyramids of the cheiroptera (bats) and rodentia, belong to the first type.

It would, however, lead to serious error, for reasons partly stated before, to attempt a classification of the pyramid tract, grounded on external characters alone. Transverse sections should be made to show the extent and deep relations of that fibre mass whose plastic elevation constitutes the so-called pyramids. Since the study of the human brain has been greatly advanced within the past decade, and constitutes from the unanimity of observers as to its most important features, as well as its representative character,* the best standard of comparison, let us proceed to lay down some of the primary facts which are to aid us in this inquiry.

### IV.—Deep Relations.

The medulla oblongata, on being cut transversely to its long axis, is found to exhibit a sort of butterfly pattern. Its two halves are united by a strand of "up-and-down fibres" known as the *raphe.* The nuclei of certain cranial nerves lie at the floor of the Fourth Ventricle (H, G, V.). From one of these, the hypoglossal nucleus (H, h) the beautiful sweep of the roots of the hypoglossal nerve can be traced, ventrad, to emerge at the surface in a furrow which separates the rounded contour of the pyramid from the similarly round contour of the olive. At no distant date all that large fibre mass, represented in the figure by the black spaces between the raphe and hypoglossal roots, was supposed to be the pyramids.† Now, however, only the part included between the lowest one-quarter of the hypoglossal and the lowest sixth of the raphe is designated as the pyramid tract (P). Immediately dorsad (above) that is, lying between the olive and the raphe, is the inter-olivary tract (L). The remainder of the field included between the raphe and the hypoglossal root, is usually known as the internal division of the "reticular field." Those of its fibres which are nearest the hyperglossal nucleus, are a continuation from the posterior longitudinal fasciculus of the tegmentum. It is thus seen that the once unified pyramid is in reality composed of four super-

* The cat, originally suggested by Wilder, may take this place some day, particularly as this animal, of all those on which the atrophy method can be tried, is nearest the human type. But for the present this is not feasible.

† Even by so recent an authority as Henle.

Transverse Section of Oblongata at the Middle Olivary Plane.

The ganglionic substances are represented by a red color; the conducting strands divided in the section by black areas; those running in the plane of the section, by white lines.

imposed strata which, if we begin near the floor of the ventricle and dig through the oblongata, would be exposed in the following order:

1. Posterior longitudinal fasciculus of the tegmentum.
2. Internal division of the reticular field.
3. Inter-olivary layer.
4. Pyramid tract (properly so-called).

In properly hardened specimens it is not difficult to distinguish the pyramid tract, as its color is slightly different from the other parts, and there is often a well-marked connective septum, or a gray intercalation belonging to the arciform nuclei, to indicate the separation.

In all the primates, the development of the pyramid tract is proportionate to the intellectual rank, and progresses quite evenly with the olivary body. Taking the area of one-half of the oblongata as 100, the following is the proportion borne by the area of the pyramid immediately above the decussation:*

| | |
|---|---|
| Man | 30 |
| Chimpanzee | 23 |
| Cebus | 10 |

Intellectual rank, or what is the anatomical index of such rank; preponderance of the highest nerve centres is not the only factor determining the size of the pyramids. The dimension of the animal has undoubtedly some influence. Thus, a large mastiff has a larger pyramid, proportionately, than a small terrier; a lion similarly exceeds the cat in this respect, and the bear the coatimundi. In drawing inferences, therefore, the size of the animal must not be disregarded. Something similar is to be said of the olivary bodies. The main nucleus of them is a simple U in the marmoset; it shows two convolution (denticulations) in a cebus; five in the baboon; twenty in the chimpanzee, and sometimes thirty in man. But

* Spitzka—"The Peduncular Tracts of the Anthropoid Apes." *Journal of Nervous and Mental Diseases*, July, 1879. In this paper, which mainly includes measurements, the general conclusion is drawn that, if we were to represent the average development of the higher (hemispheric and cerebellar) tracts of the human being as 100, the chimpanzee would rank about 75, the baboon at 40, the cebus at 25, and the dog at 7½.

it must be remembered that in small animals, the cerebral hemispheres are smooth, although convoluted in larger genera of the same zoölogical order.*

As the pons is longer in the leopard than in the cat a similar rule may apply to this tract.

No inference seems so natural as the one, that if the pyramids control the motion of the extremities, they must increase in size with the elaboration of skilled limb-motion, and decrease where these motions became less skilled. The facts, however, do not seem to be in full accord with that inference. The phocidæ, or seals and sea-lions, are aberrant carnivores, whose anterior extremities have undergone involution into flippers. On land they are unable to move with any rapidity,† and their fore-legs are not employed in the hundred motions which the cat, the dog and the bear enjoy: yet the sea-lion's pyramid tract is far superior in comparative bulk to that of these animals. Indeed, it compares favorably to that of the anthropomorpha. It may be that this is referable to the fact that the sea-lion, although the peripheral disposition of its extremity motors is clumsy, has a more absolute control, *i.e.*, voluntary control of them. It is certain that the seal can be more readily trained to use its apparently helpless flippers for

---

* Where this does not obtain, it is found, so far as I am able to say, that the discrepancy in general cerebral development is very great. Thus, the seal is a much smaller animal than the sea-lion, but it has as well convoluted a brain. With this the frontal lobes exhibit a great preponderance. In fact, their redundant development is so great that the olfactory tract is crowded into the sulcus rectus, and the gyri bordering it, overlap it and meet, so as to conceal it from view. The seal is, however, a higher development of the phocidean type. I know of nothing, except the pedigree of the equidæ that seem so continuous as the series; vison, fresh-water otter, marine otter, eared seals and earless seals. The series of brains emerges imperceptibly in this series from the dolichencephalic marten, with some ursine affinities, to the brachyencephalic seal. I may say, in passing, that the appendicular lobe of the cerebellum is enormous in the latter, and imbedded in ivory-like bone, so that I am not surprised to find it unrepresented in somes figures, it having escaped the dissector.

† Luciani's claim that the dog, whose cerebellum is removed, loses his coordination in land movements, but retains his equilibrium and power of action in the water, cannot be utilized to support the claim that a cerebellum is unnecessary in aquatic animals. The seal and sea-lion (not to mention the porpoise) have most complicated and heavy cerebellar ganglia—far higher in complexity than those of the dog.

acts requiring voluntary co-ordination than the dogs or cats. They can be trained in a few days to turn the handle of a barrel organ, no slight temptation, to acquiring the necessary

Fig. 17.—Basilar view of the sea-lion's (*zalophus gillespiei*) isthmus. Th., cut surface of thalamus; II., optic tract; III., third pair; V., fifth pair; VI., VII., VII., other cranial nerves in their order and numerals; Tr., trapezium; L., lateral field of oblongata; O., olivary eminence; P., pyramid; r., roots of the twelfth pair; r', roots of the ninth, tenth and eleventh pairs.

skill, being the exquisite musical ear which these animals have. The lines of Scott:

> " Rude Heiskar's seals, through surges dark,
> Will long pursue the minstrel's bark."

are substantiated by experience and by the high development of the auditory nerve roots * and nuclei. The complicated cerebellum of the seal is probably an expression of its position as an auditory gangloin, for the restiform column which is the spinal afferent to the cerebellum is atrophic in the seals as compared with the land carnivores. This fact is opposed to Ross' theory, that the restiform is a vegetative tract. The seals have larger visceral cavities and more complicated vis-

Fig. 18.—Lateral view of the sea-lion's isthmus. Cm., cimbria (tractus pedunculis transversus); Pn., pineal body cut; Opt., anterior pair optic lobes; pOp., posterior pair post-optic lobes; Tgtbr., anterior cerebellar peduncle, tegmentabrachium; V 4, fourth ventrical; Tub. R., tubes of rolando; Lem. lemniscus, pons, pons varolii; other references same as in preceding figure.

---

* The porpoise, whose auditory nerve is nearly double the size of the human, has probably the most acute sense of hearing in the animal kingdom. Mr. Sparks, owner of the porpoise fisheries at Cape Hatteras, informs me that his fishermen are very careful not to make any noise in laying the large nets intended to entrap them, and that porpoises have been known to change their course when an unusual noise has been made at a distance of *two miles*. He has also related to me interesting instances of *esprit de corps* among these animals, and of their high emotional nature (the porpoise will shed tears like a child when captured) which harmonise with the high cerebral development. The observations thus far made relate only to the acuteness of hearing, not to such appreciation of melody for which the seal is noted. The high development of the porpoise's brain has long been a fruitful theme for surmise among philosophers. Indeed, it was even claimed that a large brain could not be an index of mental development, because the aquatic mammalia (presupposed to be stupid) had them. The observations of an officer in our revenue marine (Capt. Charles M. Scammon, California), cera and visceral functions than the land carnivorse, but the latter have far bulkier restiform columns*. It is more probable that it can be made to harmonize with Luciani's discovery, that the aquatic movements of the dog are less interfered with than the land movements when the cerebellum is destroyed, if we remember that the restiform derivation of that ganglion preponderates in land carnivores, and the auditory derivation in aquatic carnivores.

From the forgoing it must be evident that the definition of the pyramid tract as it existed in the minds of comparative anatomists, has been too exclusively modelled on the well-ascertained facts of human anatomy. The comparative anatomist should define these bodies as follows :

The pyramids are elevated columns of the medulla oblongata, bordering the ventral fissure of the latter, and extending from the caudal border of the Pons to the inflection of the oblongata. They are never concealed by the trapezium.

The above definition takes no account of deep structural relations, or the functions of the nerve tract, to which the pyramid of the human oblongata has given its name. Before proceeding to analyze these, let us coarsely delineate the various types of pyramids found in the mammalia who possess them.

A. This type is found in man, the chimpanzee and the ourang-

---

however show that the cetacea are very sénsitive, wary and emotional. They are warmly attached to their young, confirming the accounts of the ancients about the dolphin, regarding which the great Leuret said, "That while fiction might be mingled with them, they must contain some truth." Bastian (loc. cit.) cites observations made on porpoises at the Brighton Aquarium, supporting Leuret's surmise. I have seen but one, a bottle-nosed dolphin, in captivity at the New York Aquarium. It slept neither by day nor by night, and for three weeks raced back and forth in the large tank, till it died. I inferred that this animal, accustomed to the boundless liberty of the ocean, does not manifest its natural traits in a tank any more than a man locked in a Utica crib against his will is likely to show to advantage.

* In the porpoise the facts seem to be reversed, but until the cord of this animal has been thoroughly studied the homology of the apparent restiform column is not to be considered established. It may be an overgrown inner division of the cerebellar peduncle (Meynert). I refrain from a full discussion of the seal's isthmus, as my assistants, Drs. N. E. Brill and R. Mollenhauer, are engaged in a monographic study of the subject in my laboratory. I would merely state, as my own observation, that a new nucleus, containing small nerve cells, has been discovered in the tract of the emerging facial nerve root, and that the proportions of the oblongata and pons are quite human-like in many respects. The hypoglossal nucleus is atrophic.

outang. In short, it is characteristic of the anthropomorphæ and of the anthropidæ. In the latter, the sharpness of the termination is often concealed, as are also other boundaries, by the luxuriant development of the arciform system of zonal fibres. Individual variation is claimed by Flechsig.

Fig. 19.—Type A.

The type found in the Old and New World monkeys, excluding the marmosets and lemurs is a modification of the previous type, due to the flaring out of the pyramids caudad of the olives. (Type B, Fig. 20.)

In all true monkeys the pyramids are salient, taper backwards and decussate into the opposite lateral column. In man, the chimpanzee and ourang, they are sub-cylindrical and taper gradually backwards. In semnopithecus, ateles and allied forms, the same is noted, with the exception that there is a slight flaring out at the posterior end of the olives. Even in the marmosets (nyctipithcus and hapale) the pyramids are bold and well-marked down to the pointed spinal end.

CARNIVORA.—Decrease the surface projection of the olivary fasciculus (Fig. 17) and add a corresponding bellying to the middle third of the pyramid (Fig. 18) and you have the carnivora type!

Inasmuch as the ursidæ and sea lions have a much larger pons than the other groups, it follows that the trapezium is smaller in them. In all the carnivora the posterior (spinal) end of the pyramids is pointed, being less sharp in the viverridæ then in the others. In the region of the olivary nucleus, the pyramid widens out. This is due to the bulging influence exerted by the former, which underlies them. A powerful decussation into the lateral column can be demonstrated in all. The pyramid is entirely ventrad of the trapezium.

Fig. 20.—Type B.

MARSUPALIA.—In the opossum very distinct but small pyramids are visible. They have the typical situation in the pons, but the trapezium is so large that the slender pyramids, passing ventrad of it, undergo a sharp bend in passing to the caudal oblongata. If the brain be supposed lying with the base up, the pyramids may be compared to a stream of water coming out of a cave—in the pons—and forming a low cascade at the caudal edge of the trapezium, to descend to the lower plateau of the oblongata. The decussation I have not yet examined, but as the pyramid flattens out posteriorly and appears as if diffused into the anterior column of the cord, it cannot be a considerable one.

EDENTATA.—As clumsy as these animals appear, as rudely modelled is their oblongata, the pons, which is bold and rounded, is the only salient feature, no pyramid tract nor olivary elevation is distinctly visible. The trapezium is faintly indicated, and appears to extend uninterruptedly across. (?) In an armadillo (dasypus) there is a faint indication of a pyramid elevation, without any enlargement in its course, which ends blunt at the basilar crease. The pons, compared with the sloth, is proportionately about three-quarters its size.

VESPERTILIONIDÆ.—The pyramids of the bats are well-marked. A curious feature is that, unlike other animals with decussating pyramids, those of the bats decussate as distinct bundles, not interdigitating by fasciculi. This is the condition in the small bats of the temperate zone (vesperugo, vespertilio). In the large fruit bats (pteropus fuliginosus) a more remarkable feature is added, the pyramids do not decussate in the ventral fissure, but on the basilar face of the oblongata,* so that a distinct letter X can be seen in relief here. The bundle after decussation can be traced a considerable distance, gradually losing itself in the lateral column.

Not only is the decussation of the frugivorous bats singular in its external appearance, but the course of the pyramid bundle is found to be aberrent on cross-section. While it occupies the typical position in the crus pons and trapezial part of the oblongata, it decussates, not at the junction of the cord and oblongata, as in all other animals in whom a decussation of the pyramids occurs, but high up, immediately caudad of the trapezium, so that the crossed pyramid tract, after crossing, lies

---

* Spitzka, *Journal of Comp. Med. and Surg.*, vol. 1., 18.

in the altitude of the olive, on its outer side. The crossed pyramid tract of the bat decussates entirely on the ventral surface of the oblongata, and is then situated, not in the deeper portion of the lateral column, but at the periphery of the oblongata, ventro-mesad of the direct cerebellar tract, and abutting against the processes of the nucleus antero-lateralis (of Dean and Clarke). Probably this peculiar decussation of the pyramid tract is related to the predominence of the wing-like anterior extremities, to reach whose extended nucleus the brachial will tract crosses earlier and *en masse.* The restiform column of this animal, like the pyramid tract, is large. A section immediately below the decussation, presents a singular appearance to him who is familiar with the ordinary relations among mammals. The crossed pyramids lie at the ventro-lateral periphery of the oblongata, the roots of the twelfth pair mesad (internal to) of them, and within these the typically developed olives, whose outermost parts are occasionally perforated by the nerve-roots mentioned. The olives and hypoglossal nerve-roots extend, therefore, caudad of the decussation. The latter run sharply laterad after emerging, closely applied to the membranes of the ventral face of the oblongata.

The arciform fibres field off the crossed pyramid to plunge after the usual method, into the restiform column. In some levels the nucleus of the column of Goll appears as if appended to that of the column of Burdach. and its homology is not at all very clear.

ARTIODACTYLA (RUMINANTIA).—The brains of all these animals are very similar in their basilar aspect. That of the sheep has been carefully described by Clarke and Dean, as previously stated. These observers have not made their naked eye observations as minutely as Gall,* probably because they studied

---

* The base of the brain of the calf in Plate III. of his great work is most accurate, as compared with Bastian, and even Solly. In as far as he designates the olives incorrectly he is not at all behind the former; a writer of this decade. As I have found a distinct "fifth ventricle" (pseudocele) in a calf, this adds a second to the animals below the simiadæ, who possess it; it having been found by me and confirmed by Wilder, in the dog. I have also found *striæ* at the floor of the fourth ventricle of the cat, not homologous with those of man, but in the same situation.

the brain after it was hardened in alcohol. Alcohol, unless used in cold weather, obliterates the more delicate contours of the base of the brain. In the fresh state, or in the specimen hardened in bichromate of potash the sheep's oblongata shows a beautiful sculpturing which has escaped the artists, if not the authors of the above works, with the exception of the founder of phrenology, who, if an incautious theorizer, was a truthful and thorough observer, and—for his day—a master of encephalic technology.

The brain axis of all ruminants is physiologically speaking, inferior to that of the carnivora. In the flatness, it approximates that of the sauropsida, marsupials and edentates. The smallness of the posterior corpora bigemina (post-optici) as well as their position, presents a phase of evolution which is but slightly removed from that ascertained in the iguana and alligator by Spitzka. It has been customary to designate as pyramid the column previously referred to under that name. This column, however, except in its sub-trapezial course, is in great part due to the prominence of the internal accessory olive, to which the main olivary nucleus is added in the middle third. Most of the mass is composed of zonal fibres. On cross section, only small divisions of descending fibres are noted. These gradually decrease caudad, and it is extremely probable that a pyramid tract, in the physiological sense, exists only in so far as controlling fibres are needed for the supply of the cranial nerve nuclei, and that the locomotive mechanism, involving the function of the extremities is controlled through some other, probably a thalamic tract. Hemiplegia is the rarest of accidents in veterinary practice. Brachial diplegia—paraplegia, or panplegia of the extremities are the typical forms of paralysis in the herbivorous domestic animals.

The fibres of the trapezium in the cat, dog, lion, sea lion, bear, coatimundi, ape and man run clear of the true pyramid, as seen in cross-sections. In the calf, sheep, and all other artiodactyla examined, they encroach on the pyramid more or less. Lower down in the oblongata, the same obtains with regard to the transverse fibres of Lenhossek's so-called commissure. In the first class of animals these never encroach on the pyramids. In the last-named they always do. The following appear to the writer distinct criteria of certain zoological groups.

| PRIMATES AND CARNIVORES.—Man, apes, dogs, cats, bears, seals. | UNGULATES.—Horse, horne animals, hogs, rhinoceros, etc. |
|---|---|
| 1. Pyramids, defined throughout, *a* in their lateral boundary; *b* in their termination on cross-section. | 1. Bodies termed pyramids, defined only in their cephalic third, *a* lateral boundary is lost caudad; *b* termination vague. |
| 2. The cross-section of the pyramids, increases with the preponderance of the hemispheres. | 2. No such variation determinable. If anything the reverse is noted. |
| 3. There is always a microscopically demonstrable decussation of the pyramid bundles. | 3. There never is found such a decussation microscopically visible (though figured by several. |
| 4. There is always present an inter-pyramidal fissure in the length of the oblongata *deepening* as we go caudad. | 4. There is a faint sulcus, either equally deep throughout or *becoming obliterated*, as we pass caudad. |
| 5. In cross-section, the boundary between the pyramid bundle and the neighboring olivary fibres is distinct. | 5. It is indistinct. |
| 6. In cross-section, the true pyramid is not crossed; that is, intersected and subdivided by trapezium fibres. | 6. It is so sub-divided and intersected. |

The demarcation of the calf's pyramids is correctly rendered in Gall's Plate III. In the sheep's there are some quantitative differences. The column which is, perhaps, unified externally in the bovine animals, is distinctively sub-divided in the sheep. A slender, cylindrical bundle, which runs the entire length of the oblongata, from the post-pontile border, and is of equal diameter ($1\frac{2}{10}$ millimetres) throughout lies adjacent to the basilar sulcus. Laterad of it, a slightly wider breadth is situated; this less distinct caudad than the other, and contains the olivary elevation. The latter is so beautifully marked in bichromate of potash specimens that it appears visible in all its anfractnosities, but slightly veiled by the over-lying fibres—indeed, one detachment of the olive causes a pearl-like elevation just laterad of the margin of the latter. In addition to the two bundles which represent the single columns of the ox, there is a fine curved ridge on the lateral field which courses from the trapezium to the altitude of the basilar inflection. Regarding those of both sides together they first converge in a sweep which is convex inwards, then they diverge in a second ventral sweep, which is concave inwards. There is thus formed a lyre-shaped figure, of which the trapezium constitutes the upper cross piece, while the pseudo-pyramids and the slender mesal fasciculi form the chords.

Not being in possession of an unbroken series of microscopic sections of the sheep's or ox's brain, I am unable to declare positively that *no* fibres from the so-called pyramids enter the lateral columns of the cord. I am, however, able to assert that none do so *macroscopically*. The decussation ordinarily termed the decussation of the pyramids in the hoofed animals, is now no longer known by that name. It is regarded as the decussation of the "inter-olivary layer" (Flechsig, Spitzka and Monakow) and termed the fir-cone or piniform decussation by the writer. Clarke (*vide ante*) correctly appreciated that it was homologous with what in his day was known as the upper (cephalic) or fine bundled decussation, and which the patholo-gical observations of Meyer and Spitzka have shown to be related to sensory, and possibly muscle-sense, conduction.

There is an outline resemblance between the cross-section of the oblongata of an embryonic human being prior to the development of the pyramids, and the *adult* ungulate brain. The main stress in the evolution of the latter seems to be laid on the enlargement of the internal accessory olive, in the primates and carnivores it is the V shaped main nucleus that becomes largest and most complicated.

In the elephant, the roots of the hyperglossal nerve occasionally cut through the olive, a large proportion insinuating themselves between the external accessory olive and the main nucleus. The fibre mass, ventro-mesad of the olivary nuclei in sections, is more powerful than in the ungulata (Fig. 21), but that it is not the pyramid tract is conclusively shown by its continuity with the mesal division of the lemniscus, the absence of sagittal fibres in the caudal half of the pons, and the absence of a pyramid decussatian. In no other animal, not excluding the porpoise, do I find such a deep chasm behind the pons. This is due, first to the thickness (depth) of the transverse fibre mass of the pons proper; second, to the blunt termination of the olivary masses immediately caudad of the pons. There is thus a rapid falling off of the surface before and behind.

Fig. 21.

A section through the pons of the porpoise shows the tegmentum to be three and a-half times as high as the transverse fibre mass. This is partly due to the development of an enormous auditcry nucleus (intrinsic or subendymal nucleus, Spitzka), which is so great that the median sulcus of the fourth ventricle is sunk two-thirds of an inch, as a cleft between those of both sides. (A large mass, which in a previous article, the writer was unable to interpret with certainty, and which is remarkable for its size and ventral position, being "let in" as it were, in the situation usually occupied by the trapezium, is the extrinsic auditory nucleus). The tegmental part of the raphe is to the vertical dimensions of the transverse pons fibre mass as seven to four. The posterior longitudinal fasciculus would seem to be small unless it be regarded as distorted and lengthened parallel with the raphe. The internal reticular field of the oblongata is the largest in the animal kingdom, and appears to be recruited from one field which in the pons level lies adjacent to the raphe, and from a second, which lies laterad, and corresponds in position, to the direct thalamus fasciculus. The field adjacent to the raphe in the tegmental part of the pons is remarkable in its disposition, In all other high mammals, the proportions are those of a long, attenuated triangle; in the porpoise, of an hour-glass.

Fig. 22.

The field marked x in the diagram is the one which, according to the results obtained by Spitzka in a cat, whose thalamus was destroyed, is derived from the thalamus through the decussation of the post-commissure. That this field and the lateral (uncrossed) thalamus field should be as large in the porpoise whose thalamus is also hypertrophic, seems to indicate a relation of their fibres either to the* intricate trunk motions or the exquisite sensibility† of the porpoise. The writer inclines to the former alternative, because the fields in question pass into the anterior (ventral) columns of the cord. The

* "Neurologisches Centralblatt" (Berlin), 1885. June 1st.

† The cutaneous pain sense is exquisite. I know naught of the tactile sensibility, which in a previous communication I had, perhaps erroneously, inferred to be blunt.

Fig. 23.

absence of a crossed pyramid tract renders the lateral, and the absence of a column of Goll, the posterior column, relatively small; the great height of the thalamus field in the anterior column is responsible for the heart shape of the cord section and the greater depth of the anterior fissure of the cord, as compared with the posterior.

Ordinarily, among mammals at least, the radicles of the sixth pair (abducens nerve) closely follow the contour of the internal reticular field of the tegmentum. In a cat, in which atrophy of this field had been induced, these radicles were deflected towards the raphe, on the side of the atrophy. In the porpoise, however, the roots descend directly, leaving an appreciable interval between them and the contracted field of the hour-glass (Fig. 23). The remarkable deficiency of the latter can be, provisionally at least, attributed to naught else than the rudimentary state of the hind limbs.*

Fig. 24.

It is probably not unrelated to the absence of a pyramid that the raphe of the oblongata is attenuated ventrad, and thickens progressively dorsad (Fig. 24). In primates, carnivores, rodents and other classes, where the raphe is of equal height throughout, or if anything diminished dorsad, this is due to the derivation of its

* The porpoise enjoys considerable tail motion co-ordination, in one case utilizing this member in stirring up other animals lying in the same tank of the Brighton Aquarium. In an earlier communication I stated the porpoise to be the only animal having the dorsal fissure of the cervical cord shallower than the ventral. The same state is approached by the sea-lion, and also by some ruminants.

longest fibres from the pyramids. In some levels there *is no* raphe between the porpoise's olives, the olivary fibre "halo" fusing in the middle line.

### V.—THE PYRAMIDS OF THE RODENTIA.

In 1876 Flechsig, in his great work, called attention to the remarkable relations of the pyramids in the rodentia, particularly the muridæ. It appears (loc. cit. p. 321) that a figure of the mouse's oblongata accompanying Stieda's paper first directed his notice to this subject, and I have an indistinct recollection, which I am unable to verify by my notes or works of reference, that Flechsig himself examined the rat, and found the same condition, namely that the bodies ordinarily regarded as the pyramids of these animals *decussate into the posterior columns.* The question he raises might at first appear to be a mere paraphrase of the one hinted at by Clarke, and ventilated in this paper, about the greater resemblance of the mis-called decussation of the pyramids in the horse and ungulates generally, to the decussation of the interolivary layer in man. The decussation in the rat is from powerful colnmns, with a defined inter-pyramidal fissure, which resemble in all their relations the pyramids of the first type. The decussation besides, resembles in *character* a true pyramid decussation, being in coarse bundles, and not in the delicate strands characterizing the interolivary decussation.

Flechsig, however, starting with the relations of the spinal tracts as his criterion, sweeps away all difficulties by declaring that in rodents "the bundles exposed at the ventral face of the oblongata, on either side of the median line and near the region of the pyramid decussation, are to be regarded as the interolivary layer." After a careful review of the question and a renewed study of one of the muridæ I feel compelled to regard his interpretation as unfounded.

In all the rodents named in my list, there are two columns, one on each side of the ventral fissure. They are not covered by the (very distinct) trapezium, and unlike the ungulata, their caudal ends are sharply demarcated and of definite and pointed termination. On examining a series of transverse sections *

* A complete series was prepared in my laboratory bv my assistant, Dr. Mollenhauer, with special reference to this inquiry.

of a rat's isthmus, I found that in the pons and oblongata these columns and their prolongations have exactly the same relation as in the cat, dog and other carnivora.

| | RELATION TO THE DECUSSATION. | RELATIONS IN THE OBLONGATA. | RELATIONS TO THE TRAPEZIUM-FIBERS. | RELATIONS IN THE PONS VAROLII. | RELATIONS IN THE CRUS CEREBRI. |
|---|---|---|---|---|---|
| Pyramids in Man and Carnivora. | Pass into opposite lateral co-l'm chiefly. | Situat'd ventro-mesad of olivary nucleus; more ventrad than mesad. | Lie ventrad of the trapezium fibres. | Pierce the transverse fiber mass and are separated by an appreciable interval fr'm the *ligne de separat'n.** | Continued into the *Pes.* |
| The columns in question in the Rat. | Pass into opposite posterior co-l'n chiefly. | " | " | " | " |
| Interolivary layer in Man and Carnivores | Pass into opposite posterior column. | Situated mesad of olivary nucleus. | Lie dorsad of the trapezium fibers. | Do not pierce the transverse fiber-mass and lie dorsad of the *ligne de separation.* | Continued in the *tegmentum.* |

Here we have of five characters, four in favor of the true pyramid nature of the questionable tract of the rat, and only one in favor of Flechsig's interpretation. If the rat had no other interolivary layer, the latter view might seem less conflicting with the facts. But the rat has a true interolivary layer in the usual situation, in addition to the questionable tract. Furthermore, a few, though distinct bundles, do pass from the decussation of the latter into the opposite lateral column.

* So named by Gall. It is the sharp connective tissue intercalation, which separates the true pons—that is, the transverse fiber mass—from the tegmentum.

This refutation of Flechsig's revolutionary views does not rely on my own observation alone, for Vejas and others in their representations of the rat's and rabbit's isthmus, delineate both distinct pyramid and interolivary tracts.

The writer believes that position in the crus, pons, trapezium and oblongata should be regarded as the true criteria of the homologies of the pyramid tract. Where an anatomical structure is variable in some respects in the zoological series, we seek for its unvarying features in order to establish its homologies. So far as pyramid tracts can be identified in the animal scale, they occupy homologous relations in the crus, pons, trapezium and olivary region, but variable relations in the decussation. Consequently, we must regard the constant relations as our guide. Under this guide we find that the rodentia have true pyramids, which in some forms decussate into the posterior column, just as the frugivorous bats have true pyramids, which decussate on the surface of the brain, cephalad of the usual situation, and without any connection with the raphe; in other words, differing in every possible respect from the decussation which we have come to regard as the typical form.

The connection of the true pyramid with the posterior column in the rodents named, suggests the possible existence of a closer phylogenetic relation between the pyramid tract and the posterior columns than on first sight appears plausible. We see that one and the same tract may pass into the posterior columns or into the crossed pyramidal tract, accomplishing the former in the muridæ, and the latter in the typical forms. The decussation of the interolivary layer is to be regarded as the primitive decussation, whose path was used at a subsequent period by the, functionally-speaking, higher pyramid tract as a guide. Is it to be wondered, then, that in one animal form we should have a transition stage become permanent; that is, a tract which above the decussation is a true pyramid, and below it passes in great part into the posterior columns? Even in man and the carnivora exist fibre admixtures between the interolivary layer and the pyramid tract, not to speak of the cephalic anastomosis through the bundle which passes from the pes to the tegmentum, and whose existence I must maintain as a clearly demonstrable one against several modern writers. Perhaps the older writers builded better than they knew when they called the slender columns of Gall the "posterior pyramids." Perhaps we may yet see ourselves compelled to return to the nomenclature of Meynert, which is based on the assumption of an upper (cephalic) and a lower (caudal) decussation of the pyramids. There would then be established an interesting series of transition forms in which these two intellect tracts would be shown to present relations partly of mutual support and parallel development, partly of a vicarious character.

Aside from the morphological suggestions which this subject opens up, I feel sanguine that a renewed study of the comparative development of the larger tracts will lead to important physiological conclusions. These, I think, can be framed somewhat in this way: First proposition—the elephant and the porpoise agree in having no pyramid tract; what function is equally, or nearly so, reduced in both? Answer—Elective motion of the antibrachium and digits for any other purpose than that of ambulatory and natatory locomotion, is limited or impossible in both. From this proposition the inference would be drawn that the pyramid tract is the prehensile, *i.e.*, digital controlling motor tract. In all the other ungulata, we find that part of the pyramid tract, which decussates in the typical form, absent; for those animals, elective digital motion is also limited or impossible; so that the case of all the hoofed animals sustains the inference derived from the case of the elephant and the porpoise. But so far, the proposition is merely sustained by negative facts. Let us now examine the animals who have elective digital motion, and we find that step by step, as this motion becomes more elaborate and more subjected to the higher intelligent needs, the pyramid tract is relatively enlarged.

But we are aware that the elephant possesses a co-ordinating power, as far as maintaining the erect position, balancing himself on rolling barrels, and other feats are concerned, which in kind is very like that enjoyed by the human being. His sense of weight, pressure and resistance is also highly developed. The question then arises, has the elephant any tracts which are commensurately developed with those of man? Answer, the interolivary layer and the transverse fibre mass of the pons. Inasmuch as the function of the interolivary layer

ARMY MEDICAL LIBRARY
WASHINGTON, D. C. U. S. A.

is determined by other methods to be that of conveying the muscular sense, we should infer by exclusion that the pons fibres were concerned with the power of balancing the body in space.*

Table showing proportionate development of the brain as compared with the cord, and of the pyramid tract in the Carnivora and Primates.

| Species. † | Cubic area of the entire brain (encephalon) in cubic millimeters. The measurements made in cubic centimeters originally, and then changed to millimeters. | Square area of uppermost cervical cord in millimeters. | Proportion of square area of cervical cord to cubic area of brain in millionths. | Square area of pyramid far above the decussation in millimeters. | Proportion of square area of pyramid above decussation to the area of the cervical cord in percentage. |
|---|---|---|---|---|---|
| Man (*a*).... | 1,620,000 | 81 | 50 | 33 | 40 :100 |
| " (*b*).... | 1,015,000 | 70 | 68 | 24 | 34 :100 |
| Chimpanzee | 347,000 | $49\frac{50}{100}$ | 412 | 12 | 24 :100 |
| Sea-lion... | 488,000 | 97 | 918 | 17 | 17:100 |
| Sun Bear... | 216,000 | $46\frac{50}{500}$ | 217 | — | —— |
| Leopard.... | 95,000 | $50\frac{75}{100}$ | 534 | $1\frac{5}{10}$ | 6 :100 |
| Fox....... | 41,000 | 25 | 634 | — | —— |
| Opossum... | 10,000 | $10\frac{50}{100}$ | 1050 | — | —— |

* Although the surface extent of the pons is great in the porpoise, its depth is very slight and not oue-fifth part that of the elephant. As the pons fibres, in great part, course to the frontal lobes, the deficiency of the latter in the porpoise harmonizes with the relative atrophy of the corpus striatum and the pons fibers. Iu order to make a porpoise's hemisphere, cut away the greater part of the frontal half of the elephant's hemisphere, attenuate the remainder and tilt the whole forward.

† All measurements are taken from the hardened brain; the different specimens having been hardened in the same fluids, and preserved about the same period (5–7 years). The entire area is measured by the displacement of alcohol in a graduated cylindrical vessel; the square areas by thin glass ruled in squares.

Animals with no hind limbs (porpoises) have no column of Goll. Those with reduced hind-quarters have them of smaller dimensions (seals) than those with well-developed ones (lions and man), and the atrophy is most marked in that dorsal part of the column in which the sciatic tract is to be located.

Another example of the same kind of reasoning is afforded by the comparative study of the inner pair of those bodies which are known as corpora geniculata. And although the main facts leading us to infer their possible relation to the auditory nerve have been derived from experimental researches performed by Gudden and his pupils, yet there can be little question that an earlier careful and extended study of their comparative development would have shown the following:

1st. The corpora geniculata interna are not parallel in development with the function of vision: while the anterior corpora bigemina and the external geniculata bodies are rudimentary in the blind Syrian mouse (aspalax) and the mole, the internal bodies of the same name are represented (Forel, Goddon) in their normal proportions.

2d. The corpora geniculata interna are not parallel in development with the anterior corpora bigemina. Thus the latter are much smaller than the posterior in the seal—and the only instance so far discovered among the carnivora—the internal geniculata bodies are much larger than they. They are as large in no other animal.*

3d. The optic nerve is very small in the seal. A fact which shows that largeness of the ocular globe is not necessarily related to acuteness of vision, which is also illustrated in the case of certain crepuscular animals.

4th. The auditory and trigeminus nerves are the two preponderating cranial nerves in the seal, and it is plausible in the absence of contradictory facts, to believe that the internal geniculata body has a functional connection with either of these nerves. But, inasmuch as other animals with as power-

---

* That is of those named in the list, and not marked by asterisks. Since the porpoise has a still greater disproportion of the bigeminal bodies, the posterior being very large, it is possible that still larger inner geniculate bodies occur with this species, in harmony with the observed fact that they keep step in development throughout the mammalia.

ful, or nearly as powerful, trigeminus nerves, show no commensurate development of this body, by exclusion we may regard it as an auditory ganglion.

This theory, which was within the reach of earlier comparative anatomists, but which they failed to recognize, receives support of a far stronger character from the followers of Gudden's method. They have shown that this body is directly connected with the auditory field of the cerebral cortex in dogs (V. Monakow) and the fascicular mass connecting them, already imperfectly known to Gratiolet, is found on the ventral part of the posterior-most part of the internal capsule. It is in perfect harmony with this, that the region of the seal's cerebrum, which corresponds to the dog's auditory field, is so hypertrophic that the horizontal branch of the sylvian fissure is crowded cephalo-caudad, as in no analogous type. As also in no other thoroughly examined form, a beautiful fluted fasciculus is seen on the ectal and dorsal border of the internal geniculate body. Since the auditory nerve, the inner geniculate body, the coronal bundle from the latter, and what homology suggests as the auditory temporal field, are all extraordinarily well developed in the seal, the experimentally obtained facts of V. Monakow receive a strong support. At the same time, the case of the seal shows that it is only a special field of the temporal lobe that has auditory relations, for the apex of the temporal lobe is, in this animal, atrophic.

### VI.—RELATIONS TO THE HYPOGLOSSAL NERVE ROOTS.

The emerging roots of the twelfth pair are no criterion of the boundary of the pyramids. It is true that in man these roots normally pass out in the sulcus between the lateral edge of the pyramid and the olivary eminence. But we would be reduced to absurd conclusions were we to call every fasciculi lying mesad of the hypoglossal roots the pyramids, and to deny olivary eminences to those animals, who have no olive-like body ectad of them. In the subjoined list examples will be seen of every possible mode of exit on the part of the motor nerve of the tongue—not excluding one instance where its filaments lie mesad of the pyramid bundles (Pteropus). In most animals they pass ectad of the olivary eminence, if there be one, so that the latter intervenes between the roots and the pyramid. This fact had puzzled Wilder, who, limiting his examination to the surface characters, named the laurel leaf shaped olivary field of the cat the area elliptica, thus indicating a reservation as to its significance. Older writers made some wild statements on this very head. Thus, Solly, although elsewhere (*vide ante*) manifesting powers of independent observation, approves the following, cited from Cuvier's "Leçons": "The olivary bodies are very distinct in the apes: but in some carnivores, as in bears, they are not so well formed; in others, as the lynx, badger and seal, the internal border is confounded in their whole length with the pyramidal bodies, and only distinguishable by the origin * of the twelfth pair of nerves."

A series of transverse sections shows that nothing is so inconstant as the relations, not alone of the exit, but also of the intra-medullary course of the twelfth pair. However excellent guides, certain nerve roots, such as the eighth, fifth, and the lateral mixed system, are for the searcher of homologies, the hypoglossal nerve is certainly not entitled to rank among the number. To this fact the writer called attention over seven years ago, and every subsequent observation only served to strengthen the statement then made.

MAN AND APES—The roots always emerge in the furrow between the olive and the pyramid.

In their intra-medullary course they sometimes cut through the main nucleus, others passing between the main nucleus and the *inner* accessory one.

---

* Although Solly, in blind reliance in Cuvier, falls into the error of placing roots in question between the olives and pyramids (meaning actad of the latter) on page 111, he plunges into the very opposite one on page 115, where he says, in speaking of the seal, "The corpora olivaria maintain the same central position as in the porpoise, but they do not project on the surface." Any one desirous of indulging in a, psychiatrically speaking, dangerous species of metaphysical gymnastics, may endeavor to reconcile these two statements, and attempt to picture to himself the course of the nerve as depicted in both. It must be confessed, however, that as regards the unwarrantable assimilation of the seal to the porpoise, Solly does not show to any disadvantage as compared with Meynert, who writes forty years later. Even in regard to the illusion under which his draughtsman has been stated to labor with regard to the olive of the elephant, it may be said in extenuation that an imperfect line of separation, but corresponding only to a part of the olive, exists in the place indicated in so exaggerated a way.

SEAL— The roots emerge *from* the olivary eminence * itself.

SEA LION, Carnivora generally, and most mammals not otherwise specified— The roots emerge laterad of the olivary eminence.

In their intermedullary course they pass clear of but close to the *outer* accessory and main nuclei.

ELEPHANT— The roots emerge laterad of the combined olivary and inter-olivary tract elevations.

In their intra-medullary course they cut through the *outer* accessory nucleus where it represents a lamina on cross-section, and where it is round on cross-section, they imbed it as a a stone is held in a sling.

PORPOISE— The roots emerge laterad of the olivary eminence.

In their intra-medullary course they run clear of all the olivary nuclei.†

PTEROPUS— Emerges laterad of the pyramids, and mesad of the crossed pyramid bundle.

In the intra-medullary course, it cuts through the outer accessory olive.

PARROT— Emerges in a situation homologous with that of the edentate, ungulata, etc.

In its intra-medullary conrse, it occasionally cuts into the outermost edge of the olivary lamina.

---

* This is the only instance of such an origin in my experience. The difference in the surface development of the olivary eminence in the seal and sea lion is remarkable, considering the near relationship of the two groups.

† The outer (dorsal) accessory olive of the porpoise resembles that of the elephant, the main and inner (ventral) nuclei—those of the hippopotamus, being folded with closed folds. The hypoglossal nucleus is a peculiar one in location and appearance. It is insular, removed from the typical subendymal situation, and appears to correspond rather to what in my paper on the mechanism of the brain I considered the hyoid nuclear centre. In the seal the posterior (hyo-muscular ?) rootlet of the hypoglossal is relatively as large as the cephalic rootlets are slender.

In including a non-mammal in the above list I have been actuated by the following reasons: First—The parrot is the lowest vertebrate in which a true olivary body has been demonstrated.* Second—It would lead to the strange conclusion that the hypoglossal roots emerge in the lowest forms, exactly as in the highest; that is, mesad of the olives, if Clarke's mistaken homologization of the olives in birds be allowed to pass unchallenged, Clarke speaks of a scattered mass of cells as the representative in birds of the olive of mammals, and describes their situation thus: "the cells are small and not arranged in a lamina, but scattered through the sides of the anterior column." In the parrot (ara and trichoglossus) a distinct deeply staining lamina is found, not "at the side" of the anterior column, but on either side of the raphe, with which it all but comes into contact, and from which it extends ectad, parallel with the basilar contour of the oblongata. It is richly provided with such cells as characterize the inner accessory olive of mammals to which, as found in lower forms, it corresponds, both in structure, situation and relations. The scattered group of cells seen by Clarke are present in addition, but it lies ectad of the powerful hypoglossal root, exactly as in other birds, and bears the same relation to the parrot's true olive that the nucleus antero-lateralis of Clarke bears to the olive of mammals,

The resemblance between the section of the sheep's medulla represented in Clarke's figure 14 of plate XII ("Philosophical Transactions," 1858) and that of the parrot is sufficiently great to tempt the homologizer, the outline of the olivary mass, the hypoglossal nucleus, the passage of the hypoglossal nerve and the situation of the nucleus antero-lateralis is exactly the same in both. It is, therefore, remarkable that Clarke should mistake the nucleus, which he was the discoverer of (in mammals) for an olivary nucleus in birds. The facts are these: the parrots alone, of examined birds, have a true olive; all birds, including the parrot, have a nucleus antero-lateralis, which was erroneously regarded as the representative of the olive by Clarke.

---

* By the writer in several articles, beginning with the "Olivary Body in Man, Anthropoids and Lower Mammals."—*Journal of Nervous and Mental Diseases*, 1878.

It would be interesting to determine whether other talking birds have the same olivary nucleus as the parrot, in accordance with Schroeder Van der Kolk's theory, or whether, in harmony with the author's supposition, the "dawn" of olivary development coincide with the dawning prehensile sense of the parrot's claws.

### RECAPITULATION.

1. The presence of symmetrical columnar eminences on either side of the ventral sulcus or fissure of the oblongata is a characteristic feature of the mammalia as a group. While it has become customary to designate these eminences as pyramids, this designation should not commit us to regarding them as the morphological or physiological homologues of the pyramid tract of man and the carnivora. No less than three different anatomical elements may singly or in their combination produce these elevations: First, the true pyramids—man, carnivores, rodents, bats; second, the interolivary layer—elephant; third, the olives—porpoise.

2. In probably all edentates these columns are so faintly marked as to be scarcely identifiable (sloth, armadillo).

3. In the elephant and porpoise the pyramids are demonstrably absent, as an expression probably of the physiological characters of these animals.

4. Among the remaining mammalia two different types of olivary development are found, which closely follow the zoölogical subdivisions. In the first type—primates, chiroptera, carnivora, rodentia—the pyramids are bulky, and extend as coarsely demonstrable bundles, the entire length of the oblongata, and decussate as coarse bundles into the opposite half of the cord. In the second type—ungulata—they are small in proportion to the brain, do not extend as coarsely demonstrable bundles, the entire length of the oblongata, and being apparently exhausted in supplying the cranial nerve nuclei, do not decussate as bundles into the opposite half of the cord.

With those animals having typical pyramids, the cerebral portion of these tracts has the following constant characters:

*a.* It is connected with the so-called motor-fields of the cerebral hemispheres.

*b.* Runs behind the knee of the internal capsule.

*c.* Forms a part of the pes pedunculi.

*d.* Pierces the transverse fibre mass of the pons.*

*e.* Is separated by an appreciable interval from the inter-olivary layer while in the pons.

*f.* Runs ventrad of the trapezium.

*g.* Runs ventro-mesad of the true olives.

*h.* Decussates grossly into the opposite half of the spinal cord.

6. The decussation of the true pyramids exhibits three different types

| | |
|---|---|
| In these two types the decussation represents a hypertrophy of the raphe. | A. The common type, the great mass of each pyramid passing into the opposite lateral column of the cord—primates, carnivores, some rodents.<br>B. The great mass of the pyramids passes into the opposite posterior column—muridæ, cavia (less pronounced). |
| In this type the raphe has no connection with the decussation. | C. The pyramid decussates on the surface of the brain, and passes into the lateral field of the oblongata—frugivorous bats. |

7. Among animals presenting the type A the pyramids are an index of the preponderating influence of the higher centres, and this is made manifest by two factors: First—the relative area of the trans-section of the pyramid; second—the crowding of the olives from a deep mesal situation to a more lateral and superficial one.

1st—The relative area of the pyramid tract in the oblongata increases in the order of intelligence, but this is true only for animals of the first type.

2d—The prominence of the olive—partly due to its intrinsic development—becomes more marked, as we pass up in the scale of pyramid development.

---

* Inasmuch as the transverse fibre mass, through its intercalated nuclei, gives off brainward fibres, it is only the caudal half of the pons in which this criterion of the pyramid tract is to be sought for. The remarks about the elephant's and porpoise's pons relate only to this portion.

8. Among the same group of animals there are instances where the pyramids are larger with bulkier species; thus, they are proportionately greater in the lion than in the leopard, and in the leopard than in the cat; larger in the bear than in the coatimundi; in the baboon than in the cebus. This fact seems to be in relation to the greater preponderance of the isthmus, as a whole, in larger species.

9. There is no parallelism in the mammalian series between the development of the transverse fibre-mass of the pons and the pyramids. The elephant has the largest transverse fibre mass in the series, but it has no pyramids.*

10. There is no parallelism between the development of the pyramids, and the degree to which the trapezium is concealed by the pons. This concealment is complete in the elephant and partially so in the porpoise, but neither of these animals has a pyramid in the oblongata.

11. There is no parallelism between the development of the olive and the pyramid. Both are fairly developed in the above species, though the pyramids are absent. But in animals of the same type (first type, class A) there is an approach to such parallelism, in that the U-shaped nucleus dentatus is larger with larger pyramids, and becomes convoluted with the highest forms.

12. Animals with a well-developed pyramid have a wider basilar raphe than those without such; the extreme type of the latter (porpoise) having practically none.

13. It is due to the atrophy and disappearance of the pyramid tract in the series ungulata—cetacea, proboscidea—that the transverse axis of the olivary body becomes horizontal, so that the relation of the three olivary sub-nuclei, which in man is that of ectal, main and mesal, is in these animals dorsal, main and ventral. To this general rule the elephant offers an exception, owing to the vicarious development of the inter-olivary layer in the place typically occupied by the pyramid. In all ungulata the type of the olive is the same.

---

* In human monstrosities lacking the pyramids, the pons is often fairly developed as far as the transverse fibres and nuclear intersections are concerned. The same applies to the olives.

14. The true pyramid always courses, in greater part, ventrad of the trapezium; the inter-olivary layer always passes through or over the trapezium, and where the trapezium is defective, as in man, in the homologous situation, which can be easily identified.*

### BIBLIOGRAPHY.

BASTIAN, H. Charlton.—The Brain as an Organ of the Mind. New York, D, Appleton & Co. From the English edition. 1880.

BEAUREGARD.—Recherchess ur le Cerveau du Balænoptera Sibbaldii. Societé de Biologie, communiquée le 10iéme Fevrier, 1883.

BURDACH.—Vom Bau und Leben des Gehirns. Leipzig, 1819-1826.

CARUS, Karl G.—Versuch einer Darstellung des Nervensystems. Leipzig, 1814,

CLARKE, J. Lockhart.—Researches on the Intimate Structure of the Brain—Human and Comparative. First series received June 18th, 1857. Philosophical Transactions, 1858.

DEAN, John.—The Gray Substance of the Medulla Oblongata and Trapezium. "Smithsonian Contributions to Knowledge," No. 173. Aug., 1863.

EUSTACHIUS *.—Bnh. Sgfr. Albini. Explicatio Tabularum Anatomicarum Bartholomei Eustachii, etc. Leidæ, 1761.

FLECHSIG.—Die Leitungsbahnen im Gehirn und Rückenmark des Menschen. Leipzig, 1876.

FOVILLE.—Traité Complet de l'Anatomie, etc. Paris, 1844. (Unfinished).

GALL.—Anatomie et Physiologie du Système Nerveux, etc. In 4 vols. and an atlas. Paris, 1810-1819.

GUDDEN.—Neber einen bisher nicht beschriebemen Faserstrang im Gehirn der Saügethiere und des Menschen. 1 plate. "Archiv für Psychiatrie, II., p, 364.

Experimental Untersuchungen uber das Peripherische und Centrale Nervensystem, *ib.* p. 693. With three plates.

HENLE.—Handbuch der Nervenlehre des Menschen. Braunschweig, 1871.

HUSCHKE.—Schadel Hirn und Seele. Jena, 1854.

HUXLEY, Thomas.—The Anatomy of Vertebrate Animals. 1872.

---

* In his thesis, which corroborates in the main my observations in a case of secondary degeneration of the inter-olivary tract, Schrader misconceives my description of the relations of this tract to the trapezium, evidently on the assumption that it is as marked as in the lower animals, or found in more cephalic levels. The results of v. Monakow, in this respect, obtained on a cat, agree with my description.

LEURET ET GRATIOLET.—Anatomie Comparée du Système Nerveux. Paris, 1839-1859.

LUCIANI,—On the Functions of the Cerebellum. Translated from the proceedings of the Fourth Congress of the Italian Phreniatic Society, September, 1883, by Joseph Workman, M.D., of Toronto. "Alienist and Neurologist," July, 1885.

MALACARNE.*—Neuroencefalotomia. Pavia, 1791.

MEYNERT, Th.—Ueber Unterschiede im Gehirnbau des Menschen und der Säugethiere. "Mitheilungen der Wiener Anthropologischen Gesellschaft," IV., 1870.

" Von Gehirne der Säugethiere. "Stricker's Handbuch," bd. II., pp. 694-809.

" Psychiatrie. Wien, 1884.

MISTICHELLI.*—Trattato dell' Apoplessia. Rome, 1709.

MONAKOW.—Experimentelle Beiträge zur Kenntnis der Pyramiden und Schleifenbahn. "Correspondenzblatt für Schweizer Aerzte," No. XIV., 1884.

Experimentelle Untersuchungen über Hirnrindenatrophien. Neurologisches Centralblatt," No. XXII., 1883.

McFAYDEAN.—Anatomy of the Horse. 1884.

ROHON, Victor.—Untersuchungen uber den Bau eines Mikrocephalen-Hirnes. "Arbeiten des Zool. Institutes zu Wien," Tom. II., Heft. 1.

ROSENTHAL.—Eln Beitrag zur Encephalotomie. Weimar, 1815.

SANDERSON, G. P.—Wild Animals of India. Second edition. 1879.

SANTORINI.*—Observationes Anatomicæ. Venice, 1724.

SCHRADER, Adolf.—Ein Grossoirnschenkelherd mit Secundären Degenerationes der Pyramide und Haube. "Inaugural Dissertation." Halle, 1884.

SERRES.—Anatomie Comparée du Cerveau.

SOLLY, Samuel.—The Human Brain; its Structure, Physiology and Diseases, with a description of the typical forms of brain in the animal kingdom. Second edition, 1848.

SPITZKA, E. C.—The Olivary Body in Man, the Anthropoids and Lower Mammals. "Journal of Nervous and Mental Diseases," 1878. Presented before the American Neurological Association, June, 1878.

" The Peduncular Tracts of the Anthropoid Apes. "Journal of Mental and Nervous Diseases," July, 1879.

" The Sensory Tract in the Forebrain. "Chicago Medical Review," July 5, 1881.

" Architecture and Mechanism of the Brain.' Ibid. 1879-1880.

" A Contribution to the Morbid Anatomy of Pons Lesions. "Am. Journal of Neurology and Psychiatry," November, 1883.

SPITZKA, E. C.—Contributions to the Anatomy of the Lemniscus, with remarks on the Centripetal Conducting Tracts of the Brain. "New York Medical Record," Vol. XXVI., Nos. 15–18, 1884.

" Vorlaufige Mitheilung uber einige durch die Atrophie Methode erzielte Resultate. "Neurologisches Centralblatt," June, 1885.

STIEDA, Fr.—Zeitschrift für Wissenschaftliche Zoologie. Ed. XIX.

TIEDEMAN, Fr.—Icones cerebri simiarum et quorundum mammalium rariorum. Heidelberg, 1821.

TREVIRANUS.*—Biologie oder Physiologie der lebenden Natur für Naturforscher und Aerzte. Göttingen, 1802–1823.

VEJAS, Pericles.—Experimentelle Beitrage zur Kenntniss der Verbindungsbahnen des Kleinhims und des Verlaufs der Funiculi cuneati und graciles. "Archiv. für Psychiatrie," Vol. XVI.

WILDER, B. G.—The Brain of the Cat; a Preliminary Account of its Gross Anatomy. Proceedings of the American Philosophical Society. Vol. XIX., p. 524.

" AND GAGE.—Anatomical Technology. New York and Chicago, 1882.

WILLIS, Thomas.†—Opera omnia. Geneva, 1676.

* The author desires to call attention to an omission in the bibliography of the subject indicated in the title of this paper. In Vol. V. of the "Archiv. für Psychiatrie," Westphal describes a case of *ascending degeneration* of one interolivary layer; at least, so I believe, it should be regarded, as the piniform decussation was primarily destroyed.

† For several of the above references (those marked with an asterisk), I am indebted to citations from Burdach's mentioned work, and Tarini's "Adversaria Anat."

## Figures and Tables

**Fig. 1. f1:**
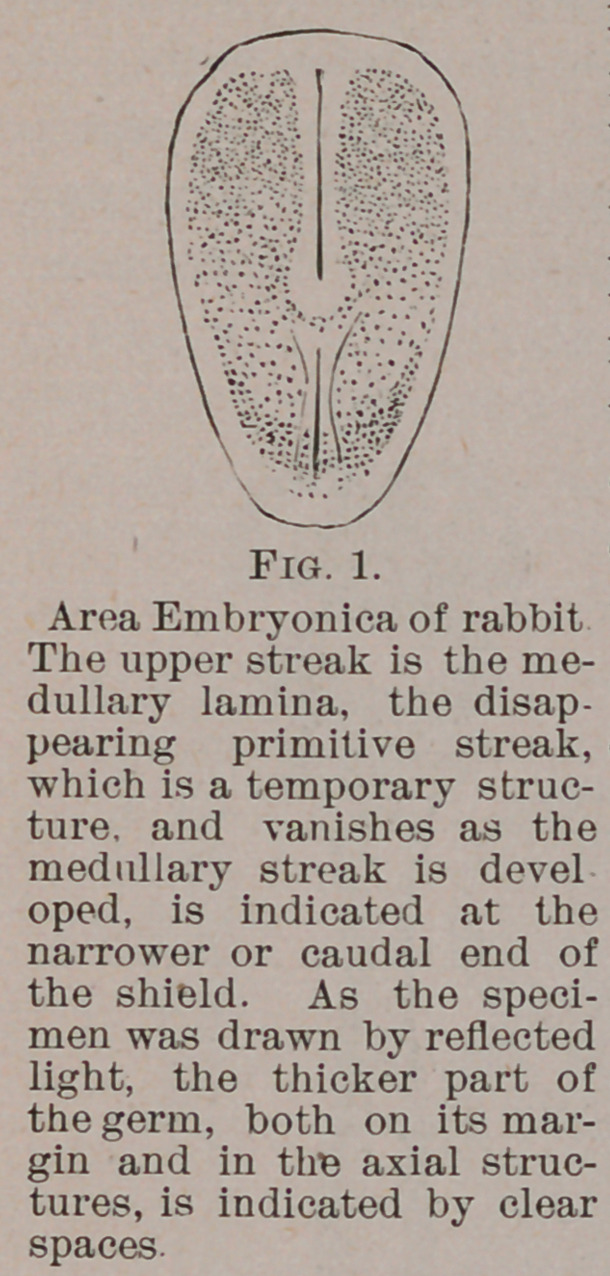


**Fig. 2. f2:**
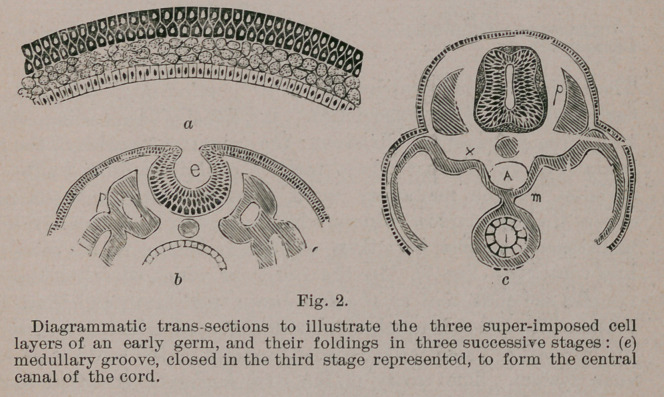


**Fig. 3. Fig. 4. Fig. 5. f3:**
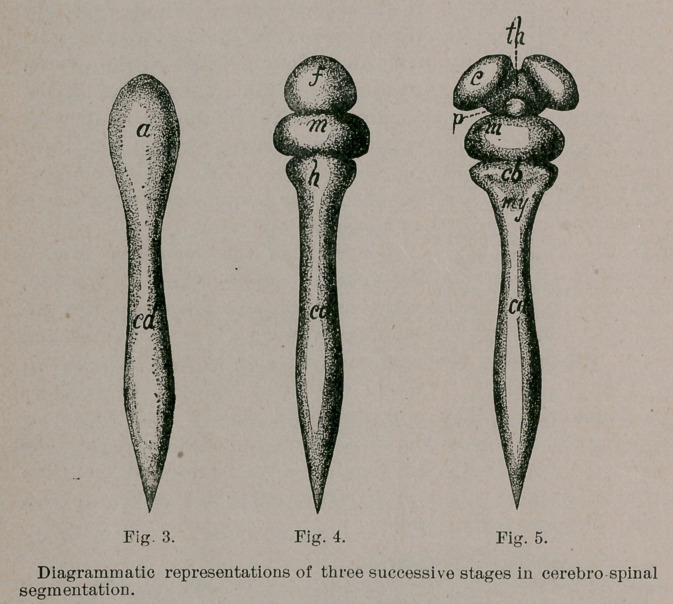


**Fig. 6. Fig. 7. Fig. 8. f4:**
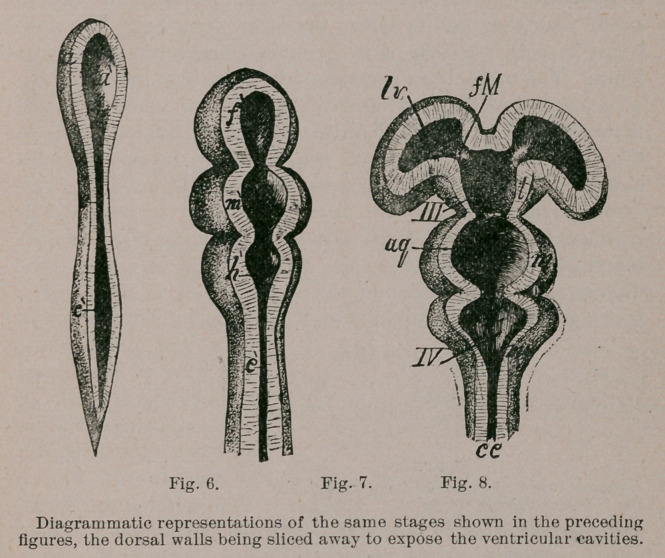


**Fig. 9. f5:**
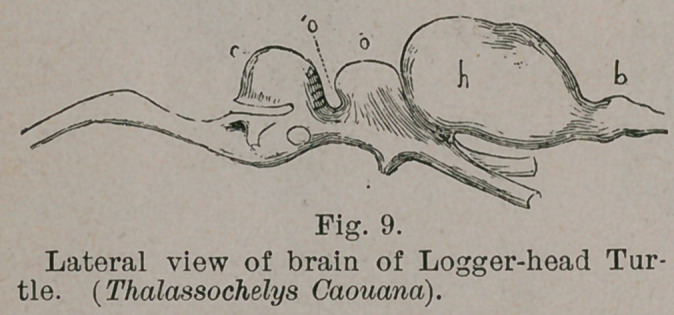


**Fig. 10. f6:**
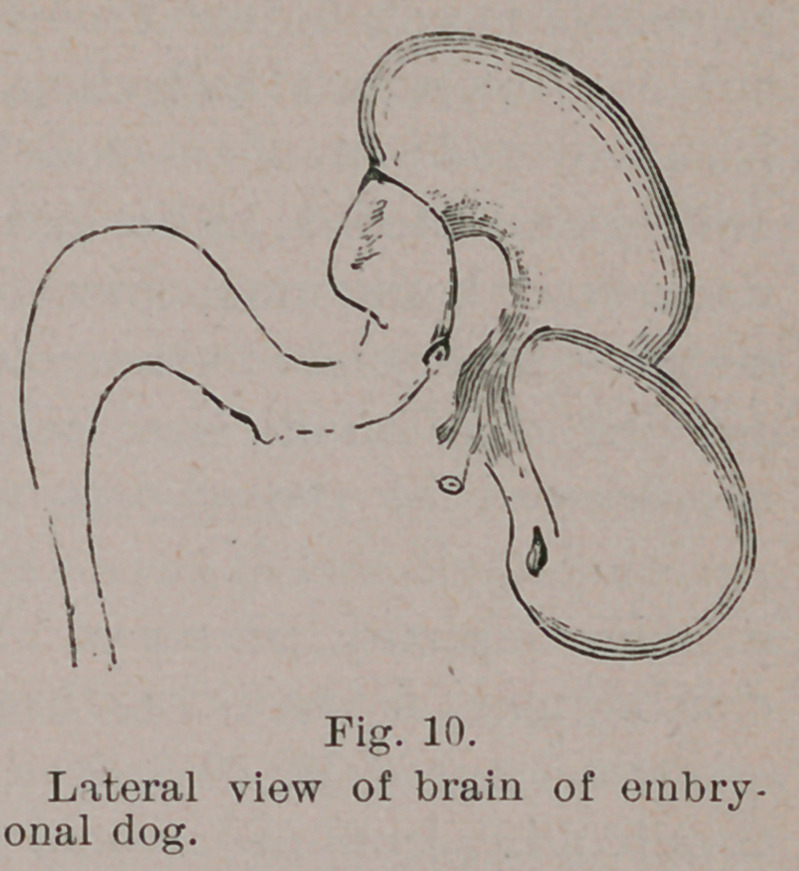


**Fig. 11. f7:**
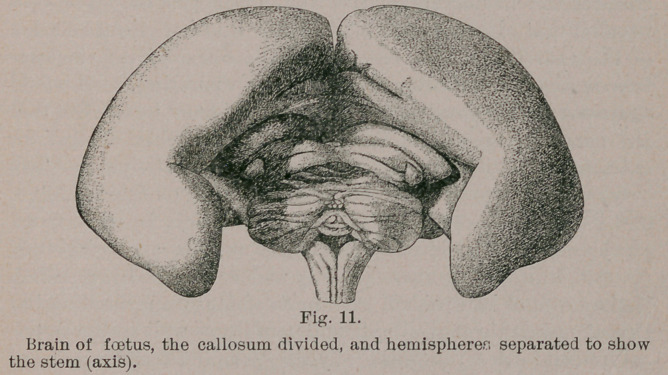


**Fig. 12. f8:**
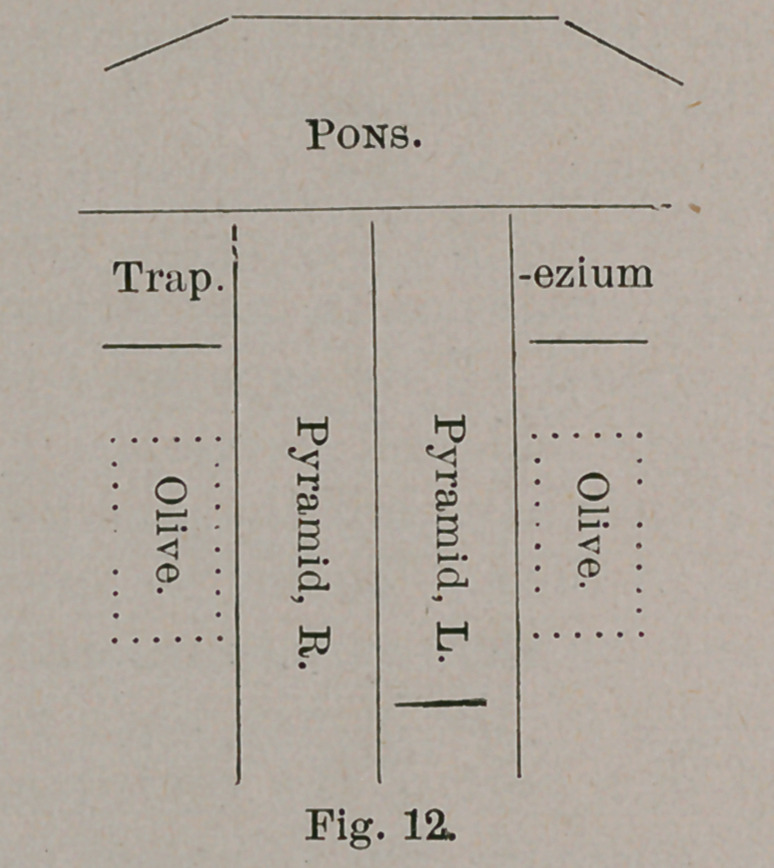


**Fig. 13. f9:**
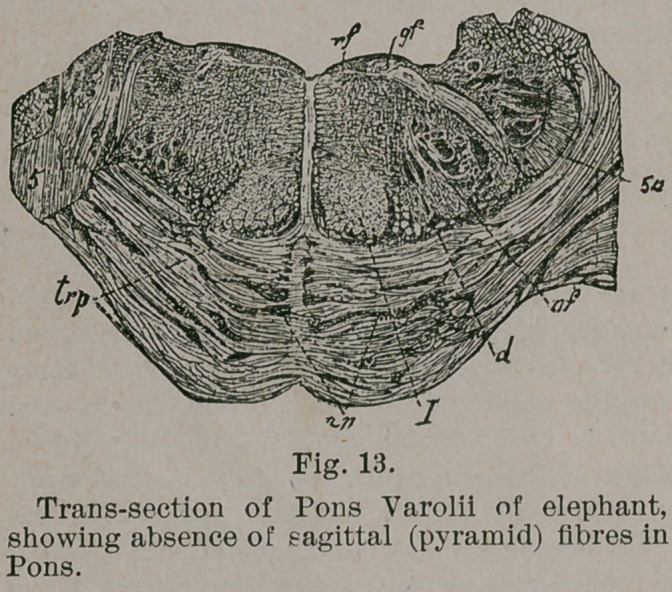


**Fig. 14. f10:**
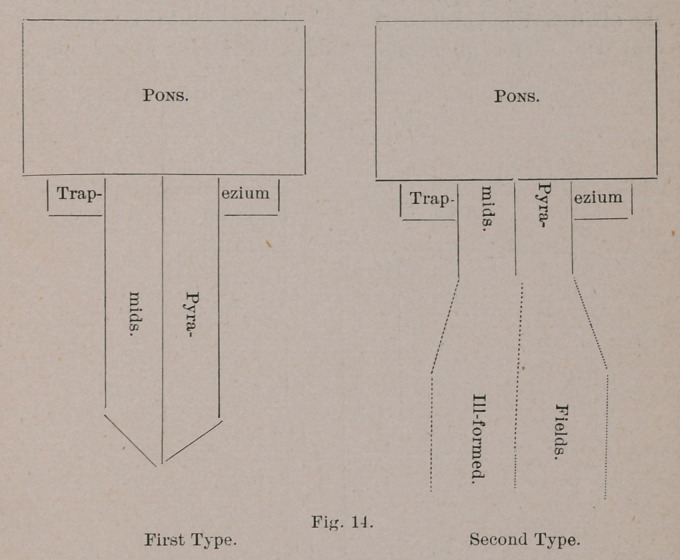


**Fig. 15. f11:**
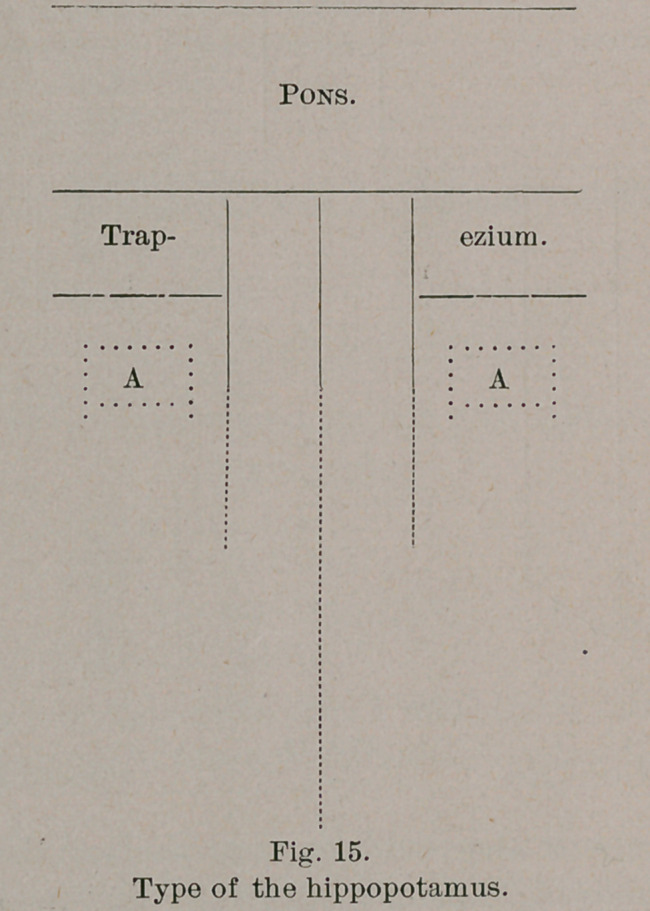


**Fig. 16. f12:**
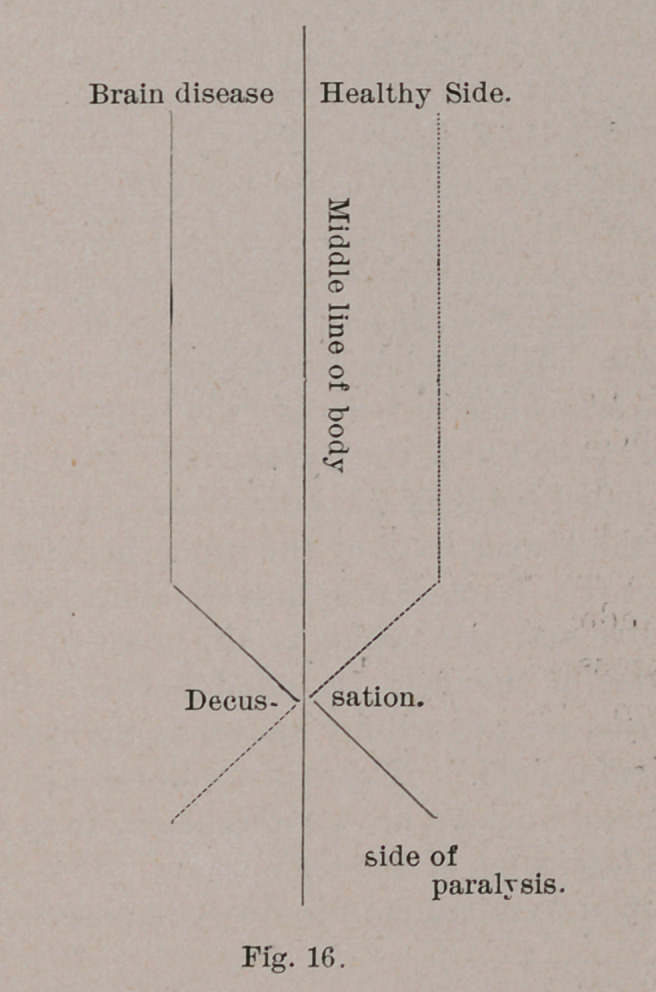


**Figure f13:**
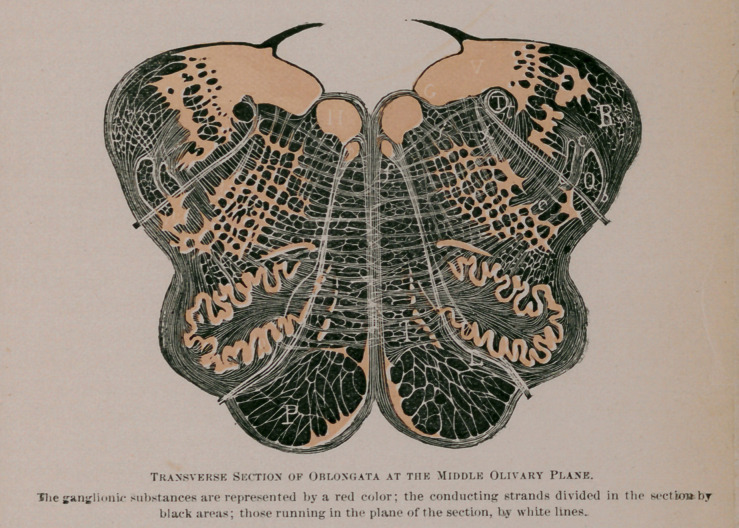


**Fig. 17. f14:**
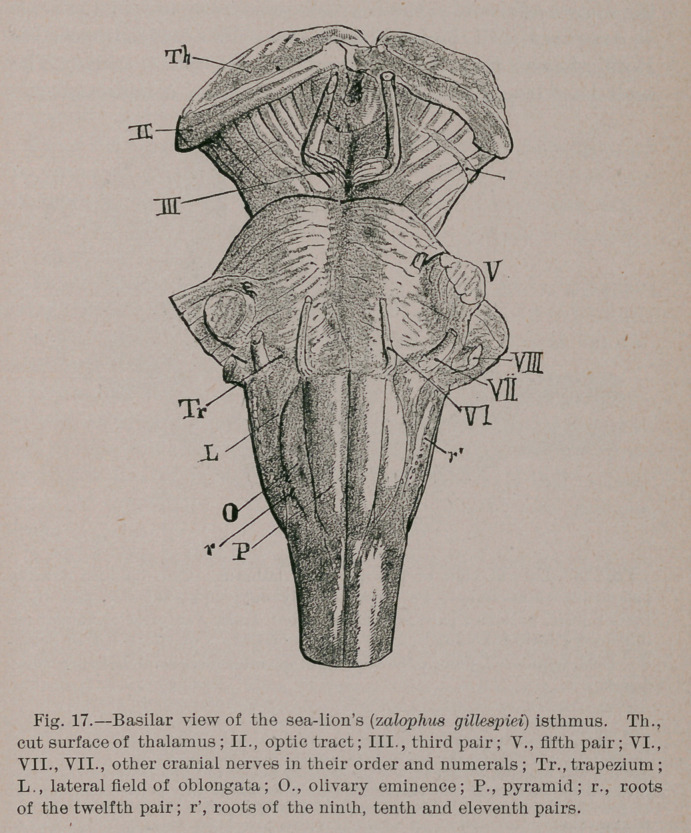


**Fig. 18. f15:**
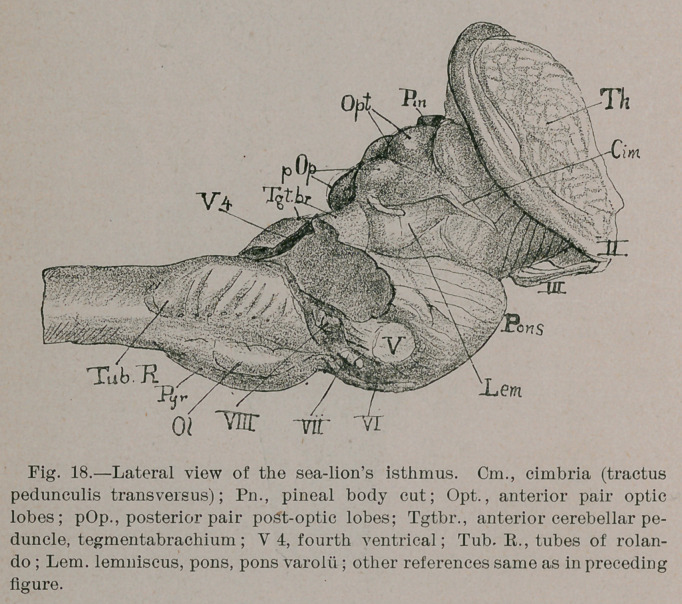


**Fig. 19. f16:**
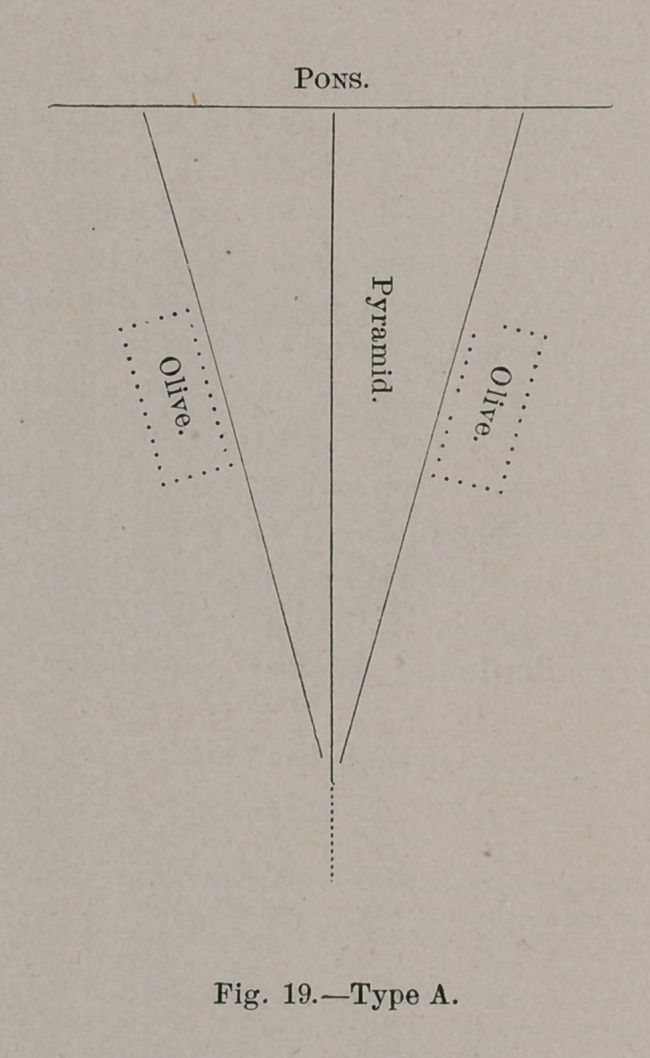


**Fig. 20. f17:**
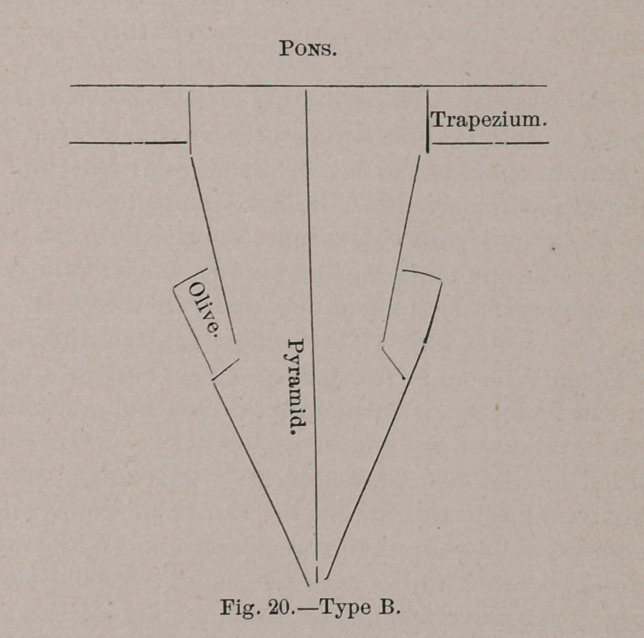


**Fig. 21. f18:**
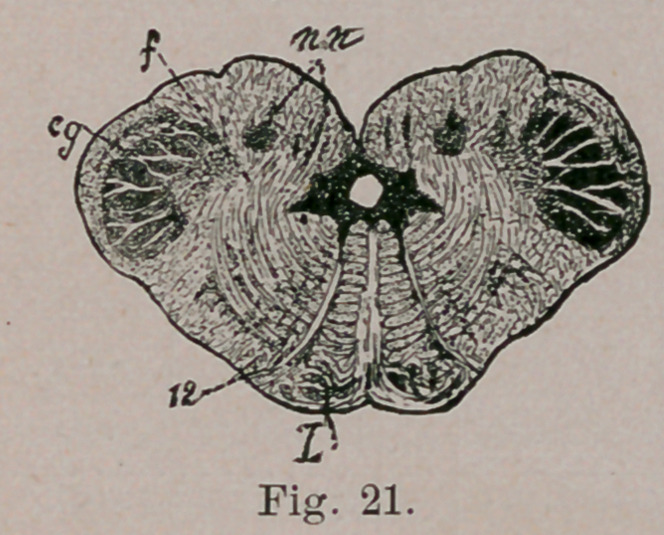


**Fig. 22. f19:**
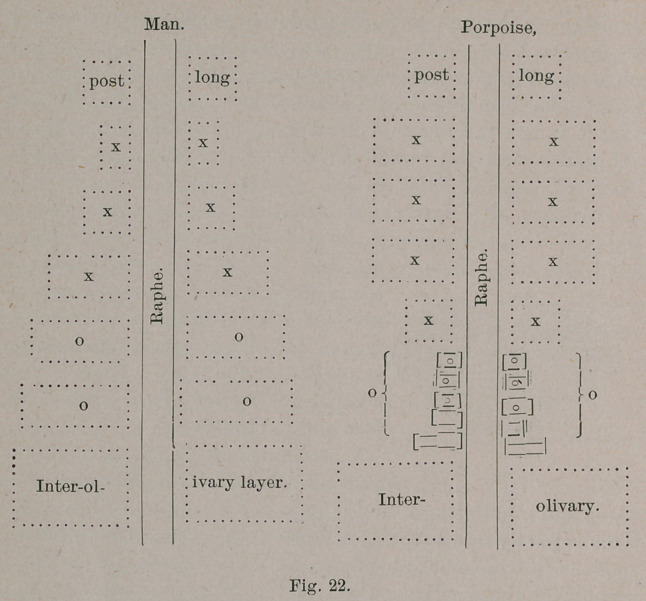


**Fig. 23. f20:**
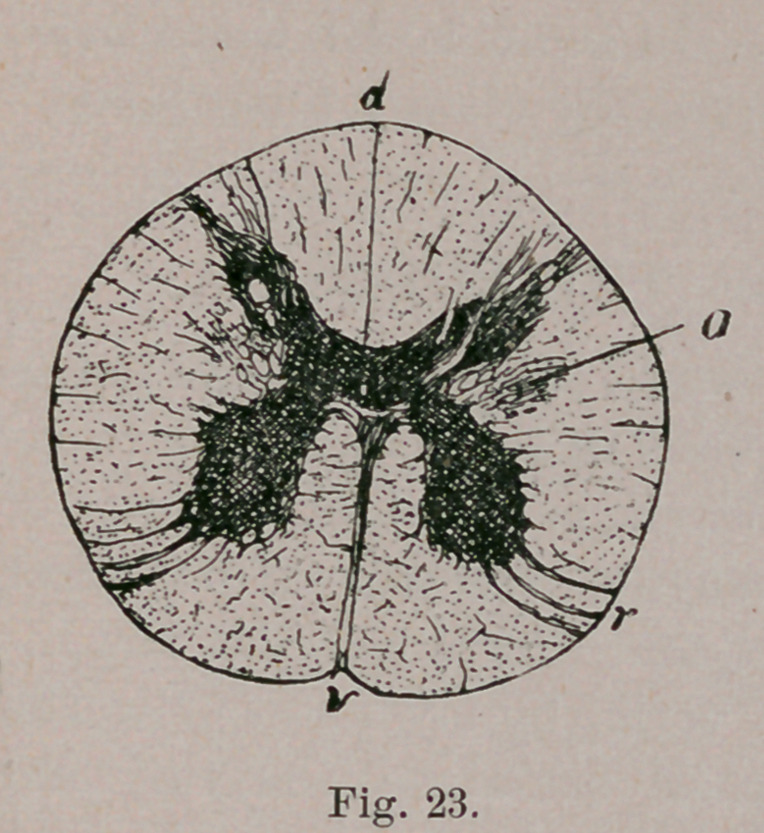


**Fig. 24. f21:**